# Multi‐Metric EEG Connectivity and Graph Analysis Reveal Altered Resting‐State Networks in Major Depressive Disorder

**DOI:** 10.1002/brb3.71842

**Published:** 2026-10-20

**Authors:** Sushmit Jahan Rose, A. H. M. Rahmatullah Imon, Rushdania Bushra, Nafis Ahmed, Moha. Tasneem Habib Warid

**Affiliations:** ^1^ Department of Computer Science and Engineering Varendra University Rajshahi Bangladesh; ^2^ Center For Interdisciplinary Research Varendra University Rajshahi Bangladesh; ^3^ Professor Emeritus of Mathematical Sciences Ball State University Muncie Indiana USA

**Keywords:** brain network analysis, functional connectivity, major depressive disorder (MDD), resting‐state EEG

## Abstract

**Purpose:**

EEG functional‐connectivity studies in major depressive disorder (MDD) have produced inconsistent findings, partly because different estimators capture distinct aspects of neural coupling. We implemented a multi‐metric resting‐state EEG approach combining linear, phase‐based, nonlinear, and graph‐theoretical measures to characterize frequency‐dependent sensor‐level connectivity alterations and identify sensor‐level network patterns relevant to depression‐related dysconnectivity.

**Method:**

Resting‐state EEG from 30 DSM‐IV MDD participants and 28 healthy controls was analyzed using a 19‐channel 10–20 montage. After MNE‐Python preprocessing, including filtering, ICA‐based artifact removal, current source density (CSD) transformation, and symmetric orthogonalization, coherence, PLV, PLI, and MI were computed across theta, alpha, and beta bands. Edge‐wise differences were tested using Brunner–Munzel with Holm correction, confirmed with Westfall–Young maxT correction, and summarized using Hedges’ *g* and graph measures.

**Finding:**

MDD showed frequency‐ and metric‐dependent sensor‐level connectivity alterations. Theta coherence, PLV, and PLI were dominated by lower connectivity across frontal‐to‐posterior, fronto‐central, temporal/posterior‐temporal, parieto‐occipital, and central‐to‐occipital sensor pairs, whereas theta MI showed few increased frontal/fronto‐temporal effects. Alpha findings were more selective, with mainly lower coherence and PLV across frontopolar/frontal‐to‐central, fronto‐midline, and posterior links, while alpha MI showed a limited mixed pattern. In beta, coherence, PLV, and PLI showed sparse robust reductions, whereas beta MI showed widespread increased statistical dependence. Graph‐theoretical summaries at 20% proportional density showed mainly lower mean weighted strength and global efficiency in theta‐ and alpha‐band networks.

**Conclusion:**

These findings indicate frequency‐ and metric‐dependent resting‐state EEG sensor‐level connectivity alterations in MDD. The dominant pattern was lower theta/alpha connectivity across distributed sensor pairs, alongside increased beta MI‐based dependence. The results provide corrected, effect‐size‐informed evidence of altered inter‐sensor coordination without establishing dysfunction of specific cortical regions or named networks. This framework may support future hypothesis‐driven research in independent, clinically annotated, higher‐density EEG or multimodal datasets.

## Graphical abstract

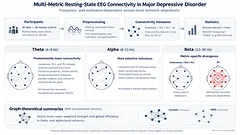

## 1 Introduction

Major Depressive Disorder (MDD) is among the most prevalent mood disorders and is characterized by persistent low mood, loss of interest, and disturbances in cognitive and affective functioning (American Psychiatric Association 2022; Cui et al. 2024). According to the World Health Organization, depression affects a substantial proportion of the global population and remains a major contributor to disability worldwide (World Health Organization 2023). Clinical assessment and self‐report scales, such as the Beck Depression Inventory‐II, remain important tools for evaluating depressive symptoms; however, they do not directly quantify neurophysiological alterations and may not fully capture the biological heterogeneity of MDD (Zimmerman 2024; Velescu et al. 2024; US Preventive Services Task Force 2023; Bradford et al. 2024). This limitation has motivated increasing interest in objective physiological approaches that can complement, rather than replace, clinical evaluation (Miljevic et al. 2023; Watts et al. 2022; Simmatis, Russo et al. 2023).

MDD is a heterogeneous disorder, and its neurobiological mechanisms cannot be reduced to a single neurotransmitter system or a single anatomical circuit. Although pharmacological and psychological treatments are effective for many patients, treatment response varies substantially across individuals. EEG offers a non‐invasive method for examining oscillatory activity and functional connectivity, but EEG‐based findings require cautious interpretation because sensor‐level recordings do not directly localize cortical generators. Therefore, rather than attempting to identify specific brain regions responsible for MDD, EEG connectivity studies can help characterize frequency‐dependent sensor‐level patterns associated with the disorder and generate hypotheses for future validation studies.

EEG has increasingly been investigated as a potential source of physiological markers relevant to MDD, including spectral features, asymmetry measures, microstate dynamics, and functional connectivity patterns (Gil Ávila et al. 2023; de Aguiar Neto and Rosa 2019; Chiarion et al. 2023). However, the clinical utility of EEG‐based biomarkers remains limited by methodological heterogeneity, differences in preprocessing strategies, variation across connectivity estimators, and insufficient external validation. Connectivity measures such as PLV and PLI have also been explored in treatment‐response contexts, including ketamine‐response prediction in MDD (Trenado et al. 2026), supporting the relevance of phase‐based connectivity analysis for depression research. Nevertheless, such findings should be interpreted as candidate markers requiring validation rather than established diagnostic or treatment‐monitoring tools.

Functional connectivity analysis provides a framework for examining statistical relationships among EEG signals across distributed sensor pairs. Different connectivity estimators capture different aspects of inter‐signal dependence. Coherence reflects frequency‐specific amplitude and phase covariance; PLV quantifies phase‐locking consistency; PLI emphasizes non‐zero‐lag phase relationships and is therefore less sensitive to volume‐conduction‐related zero‐lag effects; and MI captures more general statistical dependence, including nonlinear relationships (Chiarion et al. 2023; Kaminski and Blinowska 2022; Khaleghi et al. 2024). Because no single estimator fully characterizes EEG connectivity, a multi‐metric approach can provide complementary information while reducing overreliance on any one measure. In the present study, these estimators were applied to theta, alpha, and beta bands, and the resulting sensor‐level connectivity matrices were analyzed using a conservative edge‐wise statistical framework.

Graph‐theoretical analysis can provide secondary summaries of network organization by describing properties such as connectivity density, segregation, integration, and shortest‐path participation (Jiang et al. 2023; Chen et al. 2024; Kwon et al. 2019). In the present study, mean weighted strength, average clustering coefficient, global efficiency, local efficiency, and betweenness centrality were used as complementary network‐level descriptors; binary degree was not emphasized because proportional thresholding fixed the number of retained edges across subjects. These graph measures were interpreted as secondary summaries of the sensor‐level connectivity matrices rather than as direct evidence of source‐localized cortical network dysfunction.

Accordingly, this study applied a multi‐metric resting‐state EEG connectivity framework to a publicly available 19‐channel EEG dataset of participants with MDD and healthy controls. The analysis combined coherence, PLV, PLI, and MI across theta, alpha, and beta frequency bands, followed by edge‐wise group comparisons with formal multiple‐comparison control and effect‐size estimation. Graph‐theoretical measures were used as secondary network‐level summaries. The objective was not to establish diagnostic biomarkers or source‐localized cortical mechanisms, but to characterize frequency‐ and metric‐dependent sensor‐level connectivity alterations in MDD and to provide a transparent basis for future replication and validation studies.

## 2 Materials and Methods

### 2.1 Data Acquisition

The dataset used for this research was obtained from a previous study (Mumtaz et al. 2016). The original dataset included 33 patients diagnosed with MDD and 30 healthy volunteers from Hospital Universiti Sains Malaysia (HUSM). After preprocessing and file‐quality screening, 30 MDD participants and 28 healthy controls were included in the final analyses. MDD patients were assessed according to the diagnostic criteria for depression in the DSM‐IV, while healthy participants were examined to exclude physical and psychiatric disorders.

The EEG data were recorded during a 5‐min resting‐state eyes‐closed condition using a 19‐channel electro‐gel EEG cap system with a linked‐ear (LE) reference. The original signals were sampled at 256 Hz and amplified using a Brain Master Discovery system (Make: Brain Master; Model: Discovery 24e; Manufacturer: Brainmaster Technologies Inc.) (Mumtaz et al. 2016). Electrodes were positioned according to the International 10–20 system, including frontal/frontopolar electrodes (Fp1, Fp2, F3, F4, F7, F8, and Fz), central electrodes (C3, C4, and Cz), temporoparietal/posterior electrodes (P3, P4, Pz, T3/T7, T4/T8, T5/P7, and T6/P8), and occipital electrodes (O1, O2).

### 2.2 Data Preprocessing

All EEG recordings were preprocessed using a standardized MNE‐Python workflow (Figure 1) (Gil Ávila et al. 2023) to reduce artifacts and harmonize the data while preserving signals relevant for sensor‐level connectivity analysis. Continuous EEG data were resampled to 250 Hz to harmonize temporal resolution across recordings and reduce computational load. The continuous data were segmented into 8‐s epochs. Line‐noise contamination was attenuated using notch filtering around the power‐line frequency, followed by 1–40 Hz zero‐phase FIR band‐pass filtering to reduce slow drift and high‐frequency noise without introducing phase distortion (Figure 1).

**FIGURE 1:**
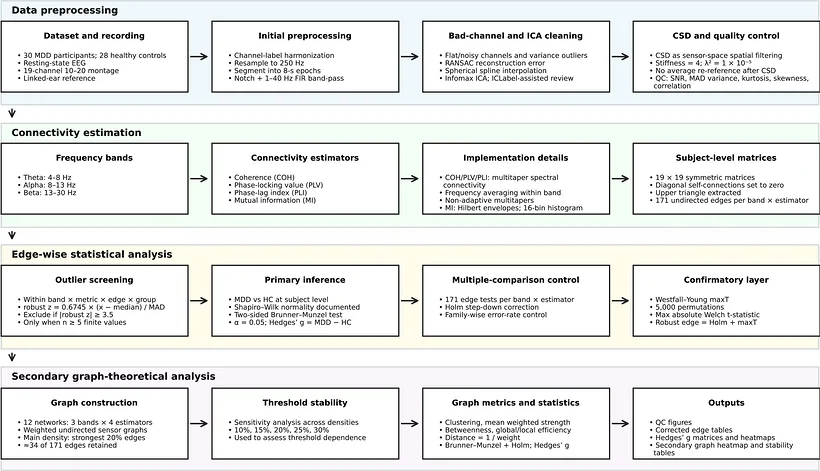
Overview of the EEG preprocessing, connectivity estimation, edge‐wise statistical testing, and secondary graph‐theoretical analysis pipeline. Resting‐state EEG data were harmonized to a 19‐channel 10–20 montage, filtered, epoched, and cleaned using bad‐channel handling and ICA, and transformed using current source density. Subject‐level connectivity matrices were estimated for theta, alpha, and beta bands using coherence, PLV, PLI, and MI. Primary edge‐wise group comparisons were performed using Brunner–Munzel testing with Holm correction and confirmed using Westfall–Young maxT permutation correction. Secondary graph‐theoretical analyses were performed on weighted undirected sensor‐level graphs using a 20% proportional density threshold, with threshold stability examined across 10%, 15%, 20%, 25%, and 30% densities.

After harmonizing the electrode labels with the international 10–20 naming convention, we assigned or approximated electrode coordinates to support consistent sensor‐space mapping. In trying to identify channels with artifacts, several criteria were used in combination: flat signals indicating potential loss of contact, variance‐based statistical outliers, and RANSAC reconstruction error to identify channels that were not functioning properly. Channels that were identified as being unreliable were dealt with in a spatially consistent manner using spherical spline interpolation. Bad‐channel interpolation was performed using spherical spline interpolation with the standard 10–20 montage coordinates and MNE‐Python's accurate interpolation mode. Channel interpolation was minimal in both groups. No interpolation was required for 27 of 28 healthy controls and 25 of 30 MDD participants. The median number of interpolated channels was 0 in both groups (HC: range 0–1; MDD: range 0–8). Detailed participant‐level channel counts and identities are provided in Table S1. Artifact reduction and removal were performed using Infomax independent component analysis (ICA). ICA dimensionality was determined by retaining components explaining 99% of the variance. For extended Infomax ICA, 1000 iterations was specified as the maximum iteration limit rather than the convergence criterion; convergence was determined by the MNE‐Python Infomax weight‐change stopping criterion using the implementation‐default threshold, as no custom convergence tolerance was specified. ICA components were classified using ICLabel for automated probabilistic identification of artifact‐related components, including ocular activity, cardiac activity, muscle activity, line noise, and channel noise (Chiarion et al. 2023). Components were excluded when ICLabel classified them as high‐confidence artifacts with probability ≥ 0.90 in one of the following categories: eye blink, muscle artifact, line noise, channel noise, or heartbeat. When ICLabel did not identify any removable component, fallback automatic artifact detection was applied using frontal‐channel ocular‐artifact detection with threshold = 3.0 and MNE‐Python muscle‐artifact detection with threshold = 0.8. The selected ICA components were excluded from the ICA solution, and the cleaned EEG signals were reconstructed using the remaining components. Because independent ground‐truth component labels were not available, formal ICLabel classification accuracy could not be estimated; therefore, preprocessing quality was evaluated using quantitative quality‐control measures before and after cleaning. Participant‐level preprocessing logs, including interpolated channels, number of interpolated channels, rejected ICA components, number of rejected ICA components, ICLabel/fallback rejection information, and retained 8‐s epochs, are provided in Table S1.

Artifacts classified as having high confidence were removed, and the cleaned data were reconstructed. To address spatial confounds, current source density (CSD) transformation was applied when electrode positions were reliable to reduce volume‐conduction effects at the sensor level; given the 19‐channel montage, this was interpreted as spatial filtering rather than precise cortical source localization (Nagy et al. 2024). In addition, regularized symmetric orthogonalization was applied after CSD transformation to minimize signal leakage and spurious correlations across channels, using a regularization strength of *λ* = 0.2·*λ*_max_, where *λ*_max_ denotes the largest eigenvalue of the channel covariance matrix (Nagy et al. 2024). No average re‐referencing was applied before or after CSD transformation. Following artifact correction and bad‐channel interpolation, CSD was used as a reference‐free sensor‐space spatial filtering step to attenuate volume‐conduction effects. Because the dataset used a low‐density 19‐channel montage, CSD‐derived spatial sharpening may be more susceptible to interpolation error than high‐density EEG; therefore, CSD results were interpreted cautiously as sensor‐level spatial filtering rather than precise cortical source localization. Spherical spline interpolation under this low spatial sampling density may also introduce spatial aliasing and signal distortion, potentially contributing to spurious sensor‐level connectivity. Although regularized symmetric orthogonalization reduces signal leakage and zero‐lag spurious correlations, it cannot correct the fundamental spatial undersampling of a 19‐channel recording. CSD was computed using a spherical spline surface Laplacian approach with stiffness = 4 and regularization *λ* ^2^ = 1 × 10^−^^5^, using the standard 10–20 electrode coordinates available in MNE‐Python. The quality of the preprocessing was objectively assessed through several quantitative indices (signal‐to‐noise ratio [SNR], robust variance from the median absolute deviation, higher‐order moments (kurtosis and skewness), and mean inter‐channel correlation) calculated before and after cleaning to document preprocessing effects and residual signal‐quality characteristics (Simmatis, Russo et al. 2023). SNR was calculated after channel‐wise z‐score normalization as:

$$\;SNR_{dB}=10\;log_{10}\;\left(\frac{P_{1-40}}{P_{45-65}}\right),$$

where *P*_1‐40_ and *P*_45‐65_ represent the mean Welch power spectral densities in the 1–40 Hz signal band and 45–65 Hz noise band, respectively.

The signal quality inspection indicated that EEGs from MDD patients presented with more variation, highly skewed amplitude distributions, and lower inter‐channel correlations than similar recordings in controls (Figure 2). These differences were treated as signal‐quality characteristics requiring transparent reporting rather than as direct evidence of disease‐specific neural mechanisms. Higher kurtosis and lower SNR‐like measures in the MDD group suggested greater artifact sensitivity or signal‐quality variability, supporting the need for transparent preprocessing and quality‐control reporting.

**FIGURE 2:**
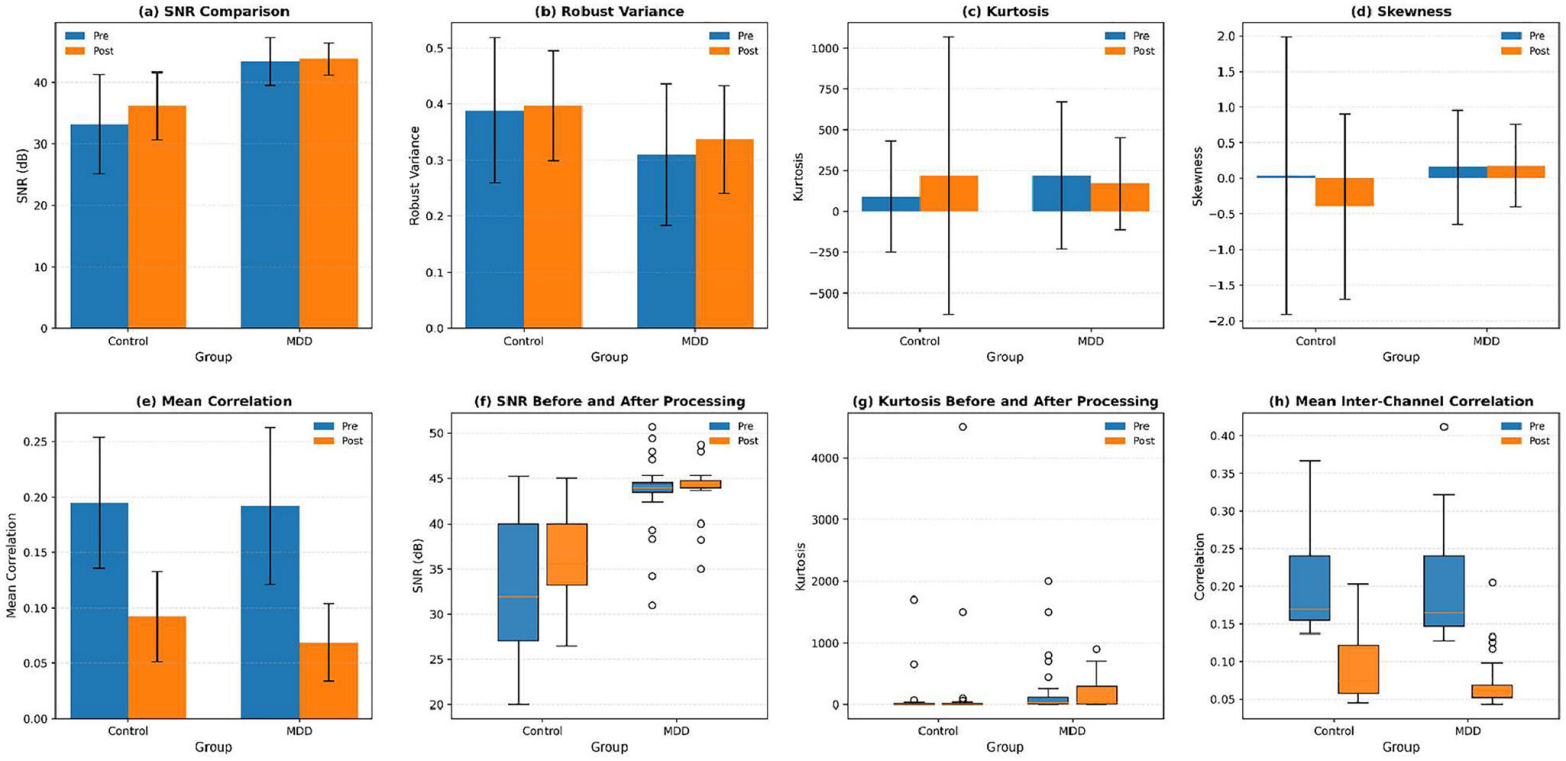
Descriptive preprocessing quality‐control summaries for healthy controls and MDD participants. Panels show group‐level summaries across preprocessing‐related quality‐control measures: (a) SNR, (b) robust variance, (c) kurtosis, (d) skewness, (e) mean inter‐channel correlation, (f) SNR improvement, (g) kurtosis reduction, and (h) inter‐channel correlation reduction. Group sample sizes were *n* = 30 for the MDD group and *n* = 28 for the healthy‐control group. Error bars/distributions summarize variability across participants. These quality‐control summaries were used to document signal‐quality characteristics and preprocessing effects and were not used to define the primary edge‐wise or graph‐theoretical findings.

After preprocessing, both groups showed improved quality‐control indices (Figure 2), including reduced variance and kurtosis, more normalized skewness, and improved inter‐channel correlation. The magnitude of change was greater for some quality‐control measures in the MDD group, suggesting that preprocessing reduced artifact‐sensitive variability in recordings with lower initial signal quality. These findings support the need for transparent quality‐control reporting in psychiatric EEG studies and confirm that preprocessing reduced several artifact‐sensitive signal‐quality measures in both groups.

### 2.3 Functional Connectivity Analysis Procedure

Functional connectivity was estimated from the preprocessed 19‐channel sensor‐space EEG data. For each subject, channels were ordered according to the common 19‐channel 10–20 montage, and fixed‐length 8‐s epochs were used for connectivity estimation. Connectivity matrices were computed separately for theta (4–8 Hz), alpha (8–13 Hz), and beta (13–30 Hz) bands (Wang et al. 2024). Four complementary connectivity estimators were applied: coherence, phase‐locking value (PLV), phase‐lag index (PLI), and mutual information (MI). Coherence, PLV, and PLI were estimated using “mne_connectivity.spectral_connectivity_epochs” with multitaper mode, frequency averaging enabled within each band, and non‐adaptive multitapers. MI was computed from band‐pass‐filtered Hilbert amplitude envelopes using a histogram‐based estimator with 16 bins. Connectivity estimates were averaged across valid 8‐s epochs within each participant to obtain one subject‐level 19 × 19 matrix for each frequency band × estimator combination. Group‐level statistical tests were therefore performed across participants, not across epochs, to avoid treating epochs from the same participant as independent observations. This multi‐metric approach was used to capture complementary aspects of linear coupling, phase synchronization, phase‐lagged interaction, and nonlinear statistical dependence (Chiarion et al. 2023; Khaleghi et al. 2024).

For each subject, band‐specific and metric‐specific 19 × 19 connectivity matrices were generated. Matrices were symmetrized, diagonal self‐connections were set to zero, and the upper triangular entries were extracted, yielding 171 undirected sensor‐level edges per band × metric family. Because the current source density transformation had been applied during preprocessing, connectivity results were interpreted as sensor‐level connectivity patterns with reduced volume–conduction influence, rather than as precise cortical source‐localized interactions (Nagy et al. 2024).

### 2.4 Statistical Analysis

Statistical inference was performed at the participant level, with each participant contributing one value per edge for each frequency band × connectivity‐estimator family. Edge‐wise group comparisons were performed at the subject level between the MDD and healthy control groups. Before inference, edge values were screened for robust outliers within each band × metric × edge × group using a median absolute deviation‐based robust z‐score procedure. Robust *z*‐scores were computed as 0.6745 × (*x* − median) / MAD, and values with |robust *z*| ≥ 3.5 were excluded from the corresponding edge‐wise test by setting them to missing. Outlier screening was performed only when at least five finite values were available within the analytic cell; when MAD was zero or non‐finite, a conventional standard‐deviation‐based *z*‐score was used as a fallback. Normality was documented using Shapiro–Wilk tests, but the primary inferential test was the two‐sided Brunner–Munzel test because normality and equal variance were not assumed. All statistical tests were evaluated at *α* = 0.05. For each band × metric family, the 171 Brunner–Munzel *p*‐values were corrected using the Holm step‐down procedure to control the family‐wise error rate (Figure 1). As a confirmatory layer, Westfall–Young maxT permutation correction was applied within each band × metric family using 5000 permutations. In this procedure, group labels were permuted and the maximum absolute Welch t‐statistic across the 171 edges was used to generate maxT‐adjusted *p*‐values. Edges surviving both Holm‐corrected Brunner–Munzel testing and Westfall–Young maxT confirmation were treated as robust findings, whereas edges surviving only the primary layer were interpreted cautiously. Effect sizes were reported using Hedges’ *g*, with direction expressed as MDD minus HC. Graph‐theoretical summaries, when reported, were derived from the same subject‐level connectivity matrices and interpreted as secondary network‐level descriptors rather than primary evidence of cortical localization (Jiang et al. 2023). These graph summaries were not used to define the primary robust edge‐wise findings.

### 2.5 Graph‐Theoretical Analysis

Graph‐theoretical analysis was performed as a secondary exploratory analysis using subject‐level 19 × 19 connectivity matrices. Graphs were constructed separately for each frequency band and connectivity estimator, yielding 12 band × estimator networks: theta‐coherence, theta‐PLV, theta‐PLI, theta‐MI, alpha‐coherence, alpha‐PLV, alpha‐PLI, alpha‐MI, beta‐coherence, beta‐PLV, beta‐PLI, and beta‐MI. For each subject‐level matrix, diagonal self‐connections were removed, and matrices were treated as weighted undirected sensor‐level adjacency matrices. For the main graph summary, the strongest 20% of edges were retained using proportional thresholding. Proportional thresholding was chosen to retain the strongest relative connections while maintaining comparable edge density across subjects and connectivity estimators. Because coherence, PLV, PLI, and MI have different numerical scales and distributions, the threshold was applied separately to each subject × frequency‐band × estimator matrix based on edge rank rather than using a common absolute connectivity cutoff. Accordingly, group comparisons were performed within each connectivity estimator rather than by directly comparing graph weights across estimators. Because each 19‐channel matrix contained 171 possible undirected edges, this threshold retained approximately 34 strongest edges per subject‐level network.

Edge weights represented connectivity strength. For shortest‐path‐based graph measures, connectivity weights were converted to graph distances using distance = 1 / weight, with a small numerical constant added to avoid division by zero. Graph metrics were computed using NetworkX and included average clustering coefficient, mean weighted strength, betweenness centrality, global efficiency, and local efficiency. Mean binary degree was not emphasized because proportional thresholding fixes the number of retained edges across subjects.

Group comparisons were performed separately for each graph metric within each band × estimator × threshold condition. The primary graph‐level comparison used the Brunner–Munzel test, and Holm correction was applied across graph measures within each band × estimator × threshold family. Effect sizes were reported using Hedges’ *g*, with direction expressed as MDD minus healthy controls. The main graph‐theoretical figure was generated at 20% density, and threshold stability was explored across proportional densities of 10%, 15%, 20%, 25%, and 30% (Figure 1). As an additional sensitivity analysis, graph measures were also computed from the full unthresholded weighted connectivity matrices. This complementary approach retained all off‐diagonal connectivity weights and therefore preserved absolute differences in connection strength without imposing equal edge density across participants. The same graph measures and group‐comparison framework were applied separately for each frequency band and connectivity estimator (Table S4). Graph‐theoretical findings were interpreted as secondary sensor‐level network summaries and were not used to define the primary robust edge‐wise connectivity findings.

## 3 Results

### 3.1 Edge‐Wise Functional Connectivity Overview

Frontopolar/frontal sensors refer to Fp1, Fp2, F3, F4, F7, F8, and Fz; central sensors refer to C3, C4, and Cz; temporal sensors refer to T7 and T8; parietal/posterior sensors refer to P3, P4, P7, P8, and Pz; and occipital sensors refer to O1 and O2. These labels are descriptive sensor‐level terms and do not imply source‐localized cortical attribution.

Across the 19‐channel montage, 171 undirected sensor‐level edges were evaluated within each band × metric family. Robust findings were defined as edges that survived both the primary Brunner–Munzel test with Holm correction and the confirmatory Westfall–Young maxT procedure. Direction was expressed as MDD minus healthy control, such that negative values indicated lower connectivity in MDD and positive values indicated higher connectivity in MDD.

At the robust layer, the overall pattern was strongly frequency‐ and metric‐dependent. Theta coherence showed 27 decreased and no increased edges, theta PLV showed 36 decreased and 1 increased edge, and theta PLI showed 49 decreased and no increased edges, indicating that theta‐band phase‐ and synchronization‐based measures were dominated by lower connectivity in MDD. Theta MI, by contrast, showed no decreased edges and three increased edges. In the alpha band, robust effects were more selective, with 9 decreased edges for coherence, 24 decreased edges for PLV, 2 decreased edges for PLI, and a mixed MI pattern comprising 1 decreased and 5 increased edges. In the beta band, coherence, PLV, and PLI retained only sparse robust reductions, whereas MI showed a marked positive shift, with 62 increased and no decreased robust edges (Table 1).

**TABLE 1:** Summary of robust edge‐wise findings by frequency band and connectivity estimator.

Metric	Theta robust edges	Alpha robust edges	Beta robust edges
Coherence	27 ↓	9 ↓	1 ↓
PLV	36 ↓ / 1 ↑	24 ↓	6 ↓
PLI	49 ↓	2 ↓	1 ↓
MI	3 ↑	5 ↑ / 1 ↓	62 ↑

*Note*: Values indicate the number and direction of robust edges surviving both Holm‐corrected Brunner–Munzel testing and Westfall–Young maxT confirmation. Direction is expressed as MDD minus healthy controls. ↓ indicates lower connectivity in MDD; ↑ indicates higher connectivity in MDD. Each cell summarizes 171 edge‐wise tests per band × estimator family in *n* = 30 MDD participants and *n* = 28 healthy controls. Robust edges met both Holm‐adjusted Brunner–Munzel *p* < 0.05 and Westfall–Young maxT‐adjusted *p* < 0.05.

### 3.2 Theta‐Band Connectivity

Theta‐band results were characterized by a broad predominance of negative Hedges’ *g* values, indicating reduced connectivity in MDD for coherence, PLV, and PLI (Figure 3). For coherence, robust reductions were concentrated across frontal‐to‐posterior, fronto‐central, temporal/posterior‐temporal, parieto‐occipital, and central‐to‐occipital sensor pairs. Representative robust edges included Fp1–O2, Fp2–Pz, F3–C4, F4–C3, F7–Cz, F8–C3, C3–O1, C3–O2, P4–O2, P8–O2, T7–O2, T8–O1, and Pz–O2. Additional reductions extended to mixed posterior and frontal‐to‐posterior pairs such as P4–T7, F4–P8, F7–P8, Fp1–P8, T7–P7, Fz–P7, and Fz–P8.

**FIGURE 3:**
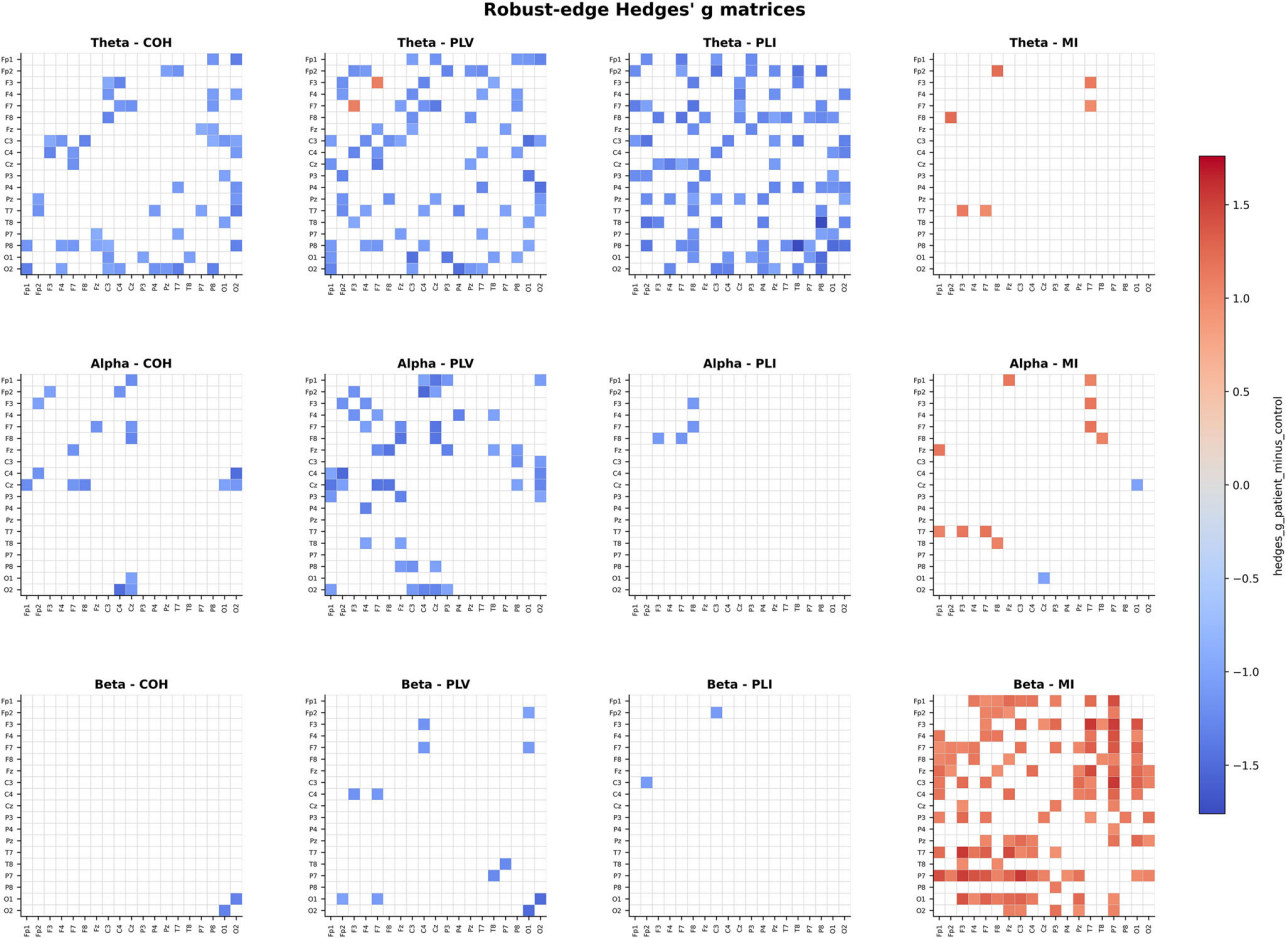
Robust edge‐wise Hedges’ *g* matrices across theta, alpha, and beta bands for coherence, PLV, PLI, and MI. Only edges surviving both Holm‐corrected Brunner–Munzel testing and Westfall–Young maxT confirmation are shown. Colors indicate Hedges’ *g* direction and magnitude, expressed as MDD minus healthy controls; blue indicates lower connectivity in MDD and red indicates higher connectivity in MDD. White cells indicate non‐robust edges, and diagonals represent self‐connections. Values above each panel show decreased/increased robust‐edge counts and total robust edges. Analyses used the final analyzable sample of 30 MDD participants and 28 healthy controls; 171 undirected edges were tested per band × estimator family using two‐sided Brunner–Munzel tests with Holm FWER correction and 5000‐permutation Westfall–Young maxT confirmation. Full edge‐level Hedges’ *g* values, *p*‐values, adjusted *p*‐values, and sample sizes are provided in Table S2.

Theta PLV showed a closely related distribution, again dominated by lower connectivity in MDD. Robust reductions involved fronto‐central, fronto‐temporal, frontal‐to‐posterior, central‐to‐occipital, and posterior‐temporal pairs. Representative robust edges included Fp1–O2, Fp2–T7, Fp2–P3, Fp2–Pz, Fp2–F3, F3–C4, F4–C3, F7–Cz, F7–P8, F8–C3, C3–O1, C3–O2, P4–O2, T7–O2, T8–O1, T7–P7, and P8–O1. Notably, theta PLV contained a single robust increase, localized to the frontal sensor pair F3–F7.

Theta PLI yielded the most extensive negative pattern of any family. Robust reductions were distributed across frontal, fronto‐central, fronto‐temporal, temporo‐parietal, parieto‐occipital, and posterior sensor pairs, indicating a widespread decrease in non‐zero‐lag phase‐based coupling. Representative robust edges included T8–P8, P8–O1, F7–F8, C3–O2, F3–F8, F4–Cz, Fz–P3, F3–T8, P4–T8, F8–Cz, Fp1–C3, P8–O2, T8–O2, Fp2–P8, T7–P8, Fp2–T8, Fp2–C3, P4–O2, F7–P8, F8–Fz, P4–O1, F4–Pz, F3–Cz, Cz–Pz, P7–O1, P3–O1, and Pz–O2.

By contrast, theta MI showed a limited but directionally opposite pattern. No robust decreases were observed, whereas three robust increases were retained, specifically at Fp2–F8, F3–T7, and F7–T7.

### 3.3 Alpha‐Band Connectivity

Alpha‐band findings were more selective than those observed in theta, with negative Hedges’ *g* values predominating for coherence and PLV, sparse reductions for PLI, and a limited mixed pattern for MI (Figure 3). Robust alpha coherence reductions were confined to nine edges and were concentrated across frontopolar/frontal‐to‐central, fronto‐midline, and central‐to‐occipital pairs. Representative robust edges included C4–O2, F8–Cz, Fp1–Cz, Fp2–C4, F7–Fz, F7–Cz, Cz–O2, Fp2–F3, and Cz–O1. Thus, alpha coherence primarily retained a circumscribed pattern of lower connectivity linking frontopolar/frontal sensors with central and posterior sensors.

Alpha PLV showed a broader but still selective negative pattern, with 24 robust decreased edges and no increases. Robust reductions were distributed across frontopolar/frontal‐to‐central, fronto‐midline, central‐to‐occipital, and frontal‐to‐posterior sensor pairs. Representative robust edges included Fp2–C4, Fp1–Cz, F8–Cz, F7–Cz, F8–Fz, F7–Fz, C4–O2, Cz–O2, Fz–P3, F4–P4, Fp2–F3, C3–P8, F3–F4, F4–F7, Fz–P8, Fp2–Cz, C3–O2, Fp1–C4, Fp1–P3, Cz–P8, Fp1–O2, P3–O2, Fz–T8, and F4–T8. Overall, alpha PLV reductions were more spatially distributed than alpha coherence reductions, but they remained notably less extensive than the corresponding theta‐band effects.

Alpha PLI was restricted to two robust decreases, both involving frontal sensor pairs: F7–F8 and F3–F8. This pattern indicated that, after confirmatory filtering, alpha PLI retained only a minimal set of reproducible reductions.

Alpha MI showed a mixed but limited pattern. Five robust increases were observed at F7–T7, F3–T7, Fp1–Fz, F8–T8, and Fp1–T7, whereas a single robust decrease was retained at Cz–O1. Accordingly, alpha MI differed from alpha coherence, PLV, and PLI by preserving both positive and negative robust effects, although the total number of survived edges remained small.

### 3.4 Beta‐Band Connectivity

As shown in Figure 3, beta‐band results showed strong metric‐specific divergence: coherence, PLV, and PLI retained only sparse negative Hedges’ *g* effects, whereas MI showed a broad positive effect‐size pattern.

For beta coherence, a single robust decrease was retained, localized to the occipital pair O1–O2. Beta PLV retained six robust decreases, specifically O1–O2, T8–P7, F3–C4, F7–C4, F7–O1, and Fp2–O1. These reductions therefore involved a limited combination of occipital, temporo‐parietal, fronto‐central, and frontal‐to‐occipital sensor pairs. Beta PLI was even more restricted, with only one robust decrease at Fp2–C3.

In marked contrast, beta MI showed 62 robust increases and no robust decreases. These increases were broadly distributed across frontopolar/frontal, central, temporal, parietal/posterior, and occipital sensors, indicating a diffuse positive shift that was not mirrored by the phase‐based beta metrics. Representative robust beta MI edges included C3–P7, F3–T7, F3–P7, Fz–T7, Fp1–P7, F7–T7, F3–O1, F7–P7, Fp1–T7, F3–P3, Fp1–C3, F7–O1, C3–O1, Fz–C4, F4–T7, Pz–O1, Fp1–F4, P3–O2, F4–P7, F7–C3, F8–O1, F3–F7, C3–T7, F8–T8, P4–P7, Pz–O2, and P7–O1. The beta MI pattern therefore differed qualitatively from all other band–metric families by combining high robust edge density with exclusively positive directionality.

### 3.5 Primary and Robust Layers

Robust findings were prioritized because they satisfied both the primary BM–Holm criterion and the confirmatory Westfall–Young maxT criterion. Several families showed complete retention of the primary layer after robust confirmation, namely theta coherence, theta PLV, theta PLI, theta MI, and beta PLV. In these families, the primary BM–Holm edges were fully preserved at the robust layer.

Other families showed partial contraction from the primary to the robust layer. Alpha coherence decreased from 10 primary edges to 9 robust edges, alpha PLV from 25 to 24, alpha MI from 7 to 6, beta coherence from 2 to 1, beta PLI from 3 to 1, and beta MI from 63 to 62. The largest contraction occurred in alpha PLI, which decreased from 6 primary edges to 2 robust edges.

Primary‐only findings were therefore interpreted cautiously. These comprised alpha coherence F8–Fz; alpha PLV F4–Cz; alpha MI C3–T7; alpha PLI edges Fp2–T8, F8–C3, Fp2–C3, and P7–O2; beta coherence F3–P4; beta PLI edges P3–P7 and P7–P8; and beta MI T7–P7. Because these edges did not survive the two‐layer criterion, they were not considered among the main reproducible findings.

### 3.6 Effect‐Size and Matrix‐Based Summary

As shown in Figure 3, the Hedges’ *g* matrices showed effect‐size patterns that matched the robust edge counts. Negative effects predominated in theta coherence, theta PLV, theta PLI, alpha coherence, and alpha PLV, whereas beta MI showed a broad positive pattern across distributed sensors. Smaller positive clusters were also observed in theta MI and alpha MI.

Overall, the edge‐wise results indicate a frequency‐ and metric‐dependent sensor‐level connectivity profile in MDD, with the strongest negative effects in theta/alpha coherence‐ and phase‐based measures and the strongest positive effects in beta MI.

### 3.7 Graph‐Theoretical Network Measures

Graph‐theoretical analysis was performed as a secondary exploratory network‐level summary using weighted undirected sensor‐level graphs. At the 20% proportional density threshold, graph‐level differences were mainly observed in theta and alpha networks, with most significant effects showing lower graph‐theoretical values in the MDD group compared with healthy controls (Figure 4).

**FIGURE 4:**
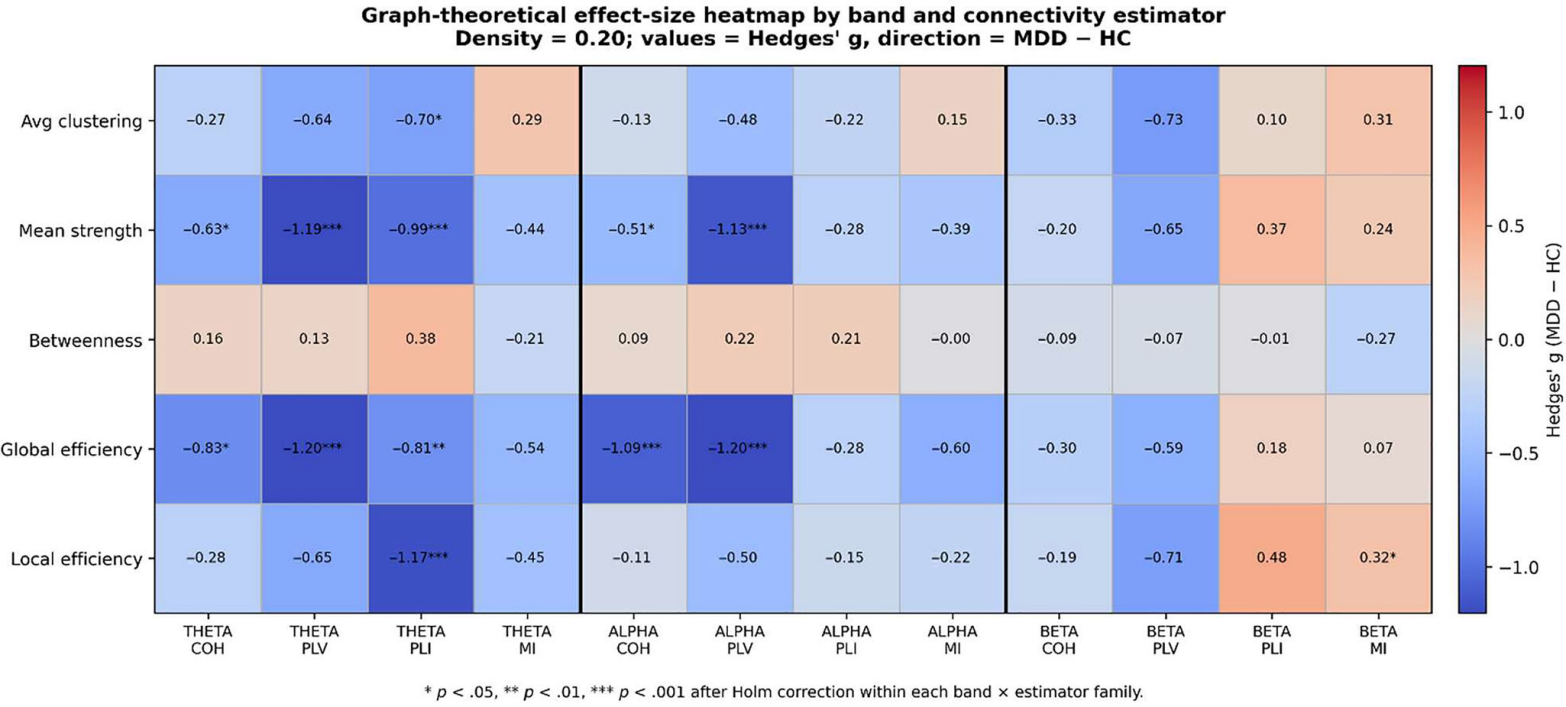
Secondary graph‐theoretical Hedges’ *g* heatmap for theta, alpha, and beta networks estimated using coherence, PLV, PLI, and MI. Weighted undirected sensor‐level graphs were constructed from subject‐level 19 × 19 connectivity matrices after retaining the strongest 20% of edges. Cell values show Hedges’ *g* expressed as MDD minus healthy controls, with negative values indicating lower and positive values indicating higher graph measures in MDD. Analyses used 30 MDD and 28 healthy‐control participants; graph metrics were compared using two‐sided Brunner–Munzel tests with Holm correction within each band × estimator family. Asterisks indicate Holm‐adjusted significance (*p_Holm_ < 0.05, **p_Holm_ < 0.01, and ***p_Holm_ < 0.001). Full graph‐level statistics are reported in Table S3.

In theta‐band networks, MDD showed lower mean weighted strength in theta‐coherence (Hedges’ *g = *−0.63), theta‐PLV (*g = *−1.19), and theta‐PLI (*g = *−0.99). Global efficiency was also lower in theta‐coherence (*g = *−0.83), theta‐PLV (*g = *−1.20), and theta‐PLI (*g = *−0.81). Theta‐PLI additionally showed lower average clustering (*g = *−0.70) and local efficiency (*g = *−1.17). In the alpha band, MDD showed lower mean weighted strength in alpha‐coherence (*g = *−0.51) and alpha‐PLV (*g = *−1.13), as well as lower global efficiency in alpha‐coherence (*g = *−1.09) and alpha‐PLV (*g = *−1.20).

Beta‐band graph‐theoretical effects were less consistent. No widespread corrected differences were observed in beta‐coherence, beta‐PLV, or beta‐PLI networks. The only Holm‐corrected beta‐band graph effect was higher local efficiency in beta‐MI in the MDD group (*g = *0.32). Betweenness centrality did not show Holm‐corrected group differences in any band × estimator network. Overall, the graph‐theoretical findings suggest reduced sensor‐level network strength and integration in MDD, particularly in theta and alpha networks.

### 3.8 Sensitivity Analysis Using Unthresholded Weighted Networks

To examine whether the graph‐theoretical findings depended on proportional thresholding, we performed an additional sensitivity analysis using full unthresholded weighted connectivity matrices. Overall, the principal network pattern was largely preserved. Across the 60 band × estimator × graph‐metric comparisons, 83.3% showed the same effect direction under both the 20% proportional‐threshold and unthresholded weighted approaches. The proportional‐threshold analysis yielded 24 Holm‐significant comparisons, whereas the unthresholded weighted analysis yielded 26, with 14 comparisons significant under both methods (Table S4). These findings indicate that the overall graph‐theoretical pattern was broadly robust to the network‐construction approach, although some metric‐specific differences remained.

## 4 Discussion

The present study examined resting‐state EEG functional connectivity in MDD using a revised sensor‐level, multi‐metric, and statistically conservative analytical framework. The main finding was not a uniform increase or decrease in connectivity, but a frequency‐ and metric‐dependent pattern of group differences. After two‐layer correction, theta‐band coherence, PLV, and PLI were dominated by lower connectivity in MDD, alpha‐band coherence and PLV showed a more selective but still predominantly negative pattern, whereas beta‐band MI showed a broad positive pattern. Thus, the findings support a model of altered sensor‐level coordination in MDD that varies according to oscillatory frequency and connectivity estimator.

A central interpretive constraint of this study is that the analysis was performed on a 19‐channel sensor‐level EEG montage. Therefore, the present findings should not be interpreted as direct evidence of dysfunction in specific cortical structures or named source‐localized networks. Electrode‐zone terms such as frontal, central, temporal, parietal/posterior, and occipital are used here only to describe the spatial distribution of sensor‐level effects. This conservative framing is important because the strongest group differences involved distributed sensor‐pair patterns rather than isolated electrodes. In particular, theta coherence, PLV, and PLI reductions involved frontal‐to‐posterior, fronto‐central, temporal/posterior‐temporal, parieto‐occipital, and central‐to‐occipital sensor pairs, including representative links such as Fp1–O2, F3–C4, F7–Cz, C3–O1, T8–P8, P8–O1, P8–O2, and Fp2–T8. These results indicate broad low‐frequency inter‐sensor differences in MDD.

### 4.1 Frequency‐Specific Interpretation

The theta‐band findings were the most extensive and internally consistent across coherence, PLV, and PLI. The predominance of lower theta connectivity in MDD is broadly consistent with prior EEG connectivity studies reporting altered low‐frequency synchronization in depression (Li et al. 2016). Theta activity has also been linked to cognitive control, monitoring, and memory‐related processes in the broader neurophysiological literature (Cavanagh and Shackman 2015; Clements et al. 2021; Ostrowski et al. 2025; Rao et al. 2025; Clouter et al. 2017; Wang et al. 2018). However, the present study cannot directly infer impairment of these cognitive systems from sensor‐level connectivity alone. Therefore, the most cautious interpretation is that MDD was associated with reduced theta‐band inter‐sensor coordination across multiple electrode zones, particularly involving anterior‐to‐posterior and posterior‐temporal/occipital sensor pairs. This pattern may be relevant to broader theories of altered network coordination in depression, but it remains hypothesis‐generating rather than mechanistic proof.

Alpha‐band findings were less extensive than theta‐band findings but followed a similar direction for coherence and PLV. Robust alpha reductions involved frontal‐central, frontopolar‐central, fronto‐midline, and posterior sensor links, including representative edges such as Fp1–Cz, Fp2–C4, F7–Fz, F8–Cz, Cz–O1, Cz–O2, Fz–P3, Fz–P8, C3–P8, and P3–O2. Alpha oscillations have been associated with inhibitory gating, attentional regulation, and flexible cognitive control (Bonnefond and Jensen 2025; Jensen 2024; Morrow et al. 2023; Segrave et al. 2011; Kiani et al. 2025; Sadaghiani and Kleinschmidt 2016). Within the limitations of the present montage, the alpha findings are therefore best framed as selective reductions in alpha‐band sensor‐level coupling, rather than direct evidence of altered inhibitory control networks. The reduced alpha coherence and PLV patterns are compatible with previous accounts of altered resting‐state organization in depression, but anatomical and functional specificity require source‐resolved or multimodal validation.

The beta‐band findings differed substantially from theta and alpha. Coherence, PLV, and PLI showed only sparse robust decreases, whereas MI showed 62 robust increases and no decreases. This distinction is critical. The result should not be summarized as generalized beta‐band hyperconnectivity, because the broad positive effect was specific to MI rather than shared across all beta‐band connectivity estimators. Prior studies have implicated beta and high‐frequency connectivity alterations in depression and related cognitive or sensorimotor processes (Lundqvist et al. 2024; Lees et al. 2025; Sihn and Kim 2024; Zhang et al. 2024). The present results are compatible with that literature, but they indicate a more specific conclusion: beta‐band MI captured broad positive sensor‐level statistical dependence in MDD, while coherence and phase‐based beta measures showed only limited robust effects.

### 4.2 Metric‐Specific Divergence and Methodological Significance

The divergence across connectivity estimators is one of the most important findings of the current analysis. Coherence and PLV showed broadly similar negative patterns in theta and alpha bands, while PLI produced the densest theta‐band reduction. This is relevant because PLI is less affected by zero‐lag coupling and volume–conduction effects than coherence or PLV, making the theta PLI findings important evidence that at least part of the theta‐band group difference reflected non‐zero‐lag phase‐based coupling rather than only common‐source contamination (Chiarion et al. 2023). This point also directly addresses the concern that EEG connectivity measures can be distorted by volume conduction.

MI behaved differently from coherence, PLV, and PLI. In theta and alpha bands, MI identified small sets of increased edges despite the predominance of negative coherence and phase‐based findings. In beta, MI showed a broad positive pattern that was not mirrored by coherence, PLV, or PLI. This does not mean that MI is contradictory or invalid; rather, it indicates that MI measures a different form of dependence. MI can capture nonlinear and distributional relationships that are not reducible to phase consistency or linear spectral covariance (Chiarion et al. 2023). Therefore, the present findings support a multi‐metric interpretation: MDD‐related EEG connectivity differences depend not only on frequency band, but also on the mathematical definition of connectivity.

The revised statistical framework also strengthens the interpretability of the results. By distinguishing primary BM‐Holm edges from robust edges that also survived Westfall–Young maxT confirmation, the analysis reduces the risk of emphasizing unstable edge‐level findings. Hedges’ *g* further provides standardized effect‐size information, while significant‐edge heatmaps and Hedges’ *g* matrices serve different purposes: the former identify correction survival and direction, whereas the latter show standardized magnitude and direction. This distinction improves reproducibility and avoids relying only on *p*‐values or visual inspection.

### 4.3 Relationship to Previous EEG and Treatment‐Response Literature

The present findings align with the broader EEG connectivity literature in suggesting that depression is associated with altered functional coupling rather than isolated regional abnormalities (Miljevic et al. 2023; Chiarion et al. 2023; Khaleghi et al. 2024; Li et al. 2016). Importantly, the present analysis extends this literature by showing that the direction and extent of MDD‐related connectivity differences depend strongly on both frequency band and connectivity estimator. The predominance of reduced theta and alpha coherence/phase‐based connectivity is consistent with prior reports of disrupted low‐frequency synchronization in depression, whereas the broad beta‐MI increase suggests that nonlinear statistical dependence may capture a different aspect of MDD‐related network alteration than coherence, PLV, or PLI. This metric‐dependent divergence may help explain why previous EEG connectivity studies in MDD have reported inconsistent findings across bands and methods.

Methodologically, these results support the value of a multi‐metric approach rather than reliance on a single connectivity estimator. Coherence, PLV, PLI, and MI did not produce interchangeable results; instead, they revealed complementary aspects of sensor‐level coordination. The current contribution is therefore not the identification of a diagnostic biomarker, but a statistically corrected and effect‐size‐informed characterization of frequency‐ and metric‐specific EEG connectivity alterations in MDD. This provides a more cautious and reproducible basis for future studies examining symptom associations, treatment response, and externally validated EEG connectivity markers. The present findings can also be situated within broader network‐level and theoretical accounts of depression. Previous graph‐theoretical and network‐based studies have reported altered brain network organization in MDD, supporting the view that depression involves distributed changes in functional coordination rather than dysfunction of a single isolated neural site (Jiang et al. 2023; Fang et al. 2021; Dai et al. 2022; Yan et al. 2023; Li et al. 2021; Zhang et al. 2022; Smit et al. 2022; van den Heuvel et al. 2024; Kaiser et al. 2025). Contemporary theoretical frameworks extend this view by conceptualizing depression as a disorder of altered information processing, network coordination, and dynamic system‐level interaction. Predictive‐coding accounts propose that major depression may involve altered weighting of predictions, prediction errors, and sensory or interoceptive evidence (Gilbert et al. 2022). Precision functional mapping has also shown altered large‐scale network topology in depression, including frontostriatal salience‐network expansion (Lynch et al. 2024). In addition, schismogenesis‐based models conceptualize mental disorders as self‐reinforcing interactions among psychological, biological, and social processes (García‐Toro and Gómez‐Juanes 2025). Although the present 19‐channel sensor‐level EEG analysis cannot directly test these models, the frequency‐ and metric‐dependent connectivity profile observed here is compatible with the broader view that MDD involves altered coordination across distributed systems.

### 4.4 Graph‐Theoretical Interpretation

At the 20% proportional density threshold, graph‐theoretical analysis showed reduced mean weighted strength and reduced global efficiency in MDD, particularly in theta‐ and alpha‐band networks. These findings indicate lower overall connectivity strength and reduced network integration in the corresponding sensor‐level networks. Theta‐PLI also showed reduced average clustering and local efficiency, suggesting that local network organization was most clearly altered in this band × estimator combination. In contrast, beta‐band graph‐level findings were less consistent, with only beta‐MI showing increased local efficiency after correction. Betweenness centrality did not show corrected group differences across the analyzed networks.

Together, the graph‐theoretical results support the edge‐wise findings by showing that the most consistent network‐level alterations in MDD were concentrated in theta‐ and alpha‐band connectivity. The pattern of reduced strength and global efficiency is consistent with previous graph‐theoretical studies reporting altered network organization in depression (Jiang et al. 2023; Fang et al. 2021; Dai et al. 2022; Yan et al. 2023; Li et al. 2021; Zhang et al. 2022; Smit et al. 2022; van den Heuvel et al. 2024; Kaiser et al. 2025). However, the dependence of graph measures on connectivity estimator and network density is important. Therefore, the 20% density threshold was used as the main graph summary, and threshold stability was examined across 10%, 15%, 20%, 25%, and 30% densities. To further evaluate the potential influence of proportional thresholding, we performed a complementary sensitivity analysis using unthresholded weighted networks, thereby preserving absolute connectivity weights without imposing equal edge density across participants. The overall pattern was broadly consistent across the two network‐construction approaches, although some metric‐specific differences remained (Table S4). Overall, the graph‐theoretical findings indicate altered sensor‐level network topology in MDD, with the clearest effects involving reduced theta‐ and alpha‐band network strength and integration.

### 4.5 Limitations and Future Directions

Several limitations constrain interpretation. First, the 19‐channel EEG montage limits spatial resolution and prevents precise source localization. Although CSD and leakage‐reduction procedures reduce spatial confounds, they do not convert the analysis into source‐space connectivity. The findings should therefore be interpreted as sensor‐level connectivity differences with reduced volume‐conduction influence, not as definitive cortical‐source interactions (Nagy et al. 2024).

Second, the study used a cross‐sectional secondary dataset. Therefore, the findings cannot establish whether the observed connectivity differences are causes, consequences, trait markers, state markers, medication‐related effects, or compensatory responses. Third, clinical variables such as symptom severity, illness duration, treatment status, medication exposure, episode structure, and comorbid symptoms were not modeled in the present analysis. For this reason, the findings should not be described as diagnostic biomarkers, predictors of treatment response, or direct targets for intervention.

Fourth, although the two‐layer correction strategy improves statistical control, the analysis still involves multiple bands, metrics, and edge families. The robust findings are conservative within the tested framework, but external replication remains necessary. Finally, MI‐based findings require additional validation because MI can be influenced by estimator settings, amplitude distributions, and preprocessing choices. The broad beta MI increase should therefore be tested in independent datasets and with alternative dependence estimators.

## 5 Conclusion

The analysis indicates that MDD is associated with frequency‐ and metric‐dependent alterations in resting‐state EEG sensor‐level connectivity. The most reproducible reductions were observed in theta coherence, theta PLV, theta PLI, alpha coherence, and alpha PLV, whereas beta MI showed predominantly increased sensor‐level statistical dependence. These findings do not support a simple global hypo‐ or hyperconnectivity model; rather, they show that group differences depend on both oscillatory frequency and connectivity estimator.

Graph‐theoretical analysis further showed reduced mean weighted strength and reduced global efficiency in MDD, particularly in theta‐ and alpha‐band networks at the 20% proportional density threshold. This pattern indicates lower sensor‐level network strength and reduced network integration in these frequency ranges, broadly supporting the edge‐wise findings.

Overall, this study provides a statistically corrected and effect‐size‐informed characterization of altered resting‐state EEG connectivity in MDD within the limits of a 19‐channel sensor‐level design. The low spatial sampling density may introduce interpolation‐related spatial aliasing and potentially spurious connectivity, and symmetric orthogonalization cannot remedy this fundamental spatial undersampling. The findings suggest that MDD is associated with altered inter‐sensor coordination across multiple oscillatory bands and connectivity estimators, while remaining limited by the sensor‐level design and the need for external replication. By combining edge‐wise statistics with graph‐theoretical summaries, this study highlights a frequency‐ and metric‐dependent connectivity profile that may guide future high‐density EEG, MEG, source‐space, multimodal, and clinically annotated studies examining the neurophysiological relevance of these patterns.

## Author Contributions

**Sushmit Jahan Rose**: conceptualization, methodology, software, data curation, formal analysis, visualization, investigation, writing – original draft, writing – review and editing. **A. H. M. Rahmatullah Imon**: validation, methodology, writing – review and editing. **Rushdania Bushra**: data curation, investigation, writing – original draft. **Nafis Ahmed**: software, visualization, data curation, writing – original draft. **Moha. Tasneem Habib Warid**: data curation, validation, writing – original draft. All authors have read and approved the final version of the manuscript and agree to be accountable for all aspects of the work.

## Funding

This research was supported by the Center for Interdisciplinary Research, Varendra University, Bangladesh.

## Ethics Statement

This study used a publicly available EEG dataset collected by Mumtaz et al. All experimental procedures in the original study were approved by the relevant institutional ethics committee.

## Consent

The data used in this study were obtained from a previously published dataset (Mumtaz et al.). All participants in the original study provided written informed consent prior to data collection. No additional consent was required for this secondary analysis, as all data were fully anonymized and publicly available.

## Conflicts of Interest

The authors declare no conflicts of interest.

## Supporting information




**Supplementary Material**: brb371842‐sup‐0001‐SuppMat.docx

## Data Availability

The dataset analyzed during the current study is publicly available from the work of Mumtaz et al. The analysis code will be made available upon request.
