# Supplementary material for: Multi‐Metric EEG Connectivity and Graph Analysis Reveal Altered Resting‐State Networks in Major Depressive Disorder
Source: Brain Behav. 2026 Oct 20;16(10):e71842. doi: 10.1002/brb3.71842 (PMC13684065; doi:10.1002/brb3.71842)
Supplement: Supplementary file 1 [file BRB3-16-e71842-s001.docx]

**Supplementary Tables**

*Tables S1, S2, S3 and S4*

**Supplementary Table S1. Participant-level preprocessing summary.**

This table summarizes the 19-channel preprocessing log generated from the EDF files. Bad/interpolated channels were detected using flat-channel, variance-outlier, and low-correlation criteria. ICA was performed using Infomax with 99% variance retention and up to 1,000 iterations. ICLabel artifact components were excluded when classified as high-confidence artifacts with probability ≥ 0.90; fallback frontal ocular-artifact detection and muscle-artifact detection were applied when no ICLabel component was selected. CSD parameters were stiffness = 4 and λ² = 1 × 10⁻⁵.

**Group summary**

| Group | n_subjects | median_interpolated_channels | IQR_interpolated_channels | range_interpolated_channels | median_rejected_ICA_components | IQR_rejected_ICA_components | range_rejected_ICA_components | n_fallback_EOG | n_fallback_muscle | median_retained_8s_epochs | range_retained_8s_epochs |
| --- | --- | --- | --- | --- | --- | --- | --- | --- | --- | --- | --- |
| HC | 28 | 0.000 | 0.00–0.00 | 0–1 | 1.000 | 1.00–2.00 | 0–4 | 1 | 9 | 37.000 | 36–47 |
| MDD | 30 | 0.000 | 0.00–0.00 | 0–8 | 1.000 | 0.00–1.00 | 0–2 | 0 | 1 | 37.000 | 22–37 |

**Per-subject summary**

| subject_id | group | condition | status | bad_channels_interpolated | n_bad_channels | ica_excluded_components | ica_exclusion_reasons | n_ica_excluded | fallback_eog_used | fallback_muscle_used | n_epochs_8s | missing_19ch_channels |
| --- | --- | --- | --- | --- | --- | --- | --- | --- | --- | --- | --- | --- |
| H S1 EC | HC | EC | ok |  | 0 | 0,1 | ICLabel:eye blink:0.980;ICLabel:eye blink:0.999 | 2 | False | False | 37 |  |
| H S10 EC | HC | EC | ok |  | 0 | 7 | ICLabel:eye blink:0.973 | 1 | False | False | 47 |  |
| H S11 EC | HC | EC | ok |  | 0 | 14 | fallback_muscle | 1 | False | True | 37 |  |
| H S13 EC | HC | EC | ok |  | 0 | 5 | ICLabel:eye blink:0.983 | 1 | False | False | 37 |  |
| H S14 EC | HC | EC | ok |  | 0 | 13 | fallback_muscle | 1 | False | True | 37 |  |
| H S15 EC | HC | EC | ok |  | 0 | 0,7,10,11 | fallback_muscle;fallback_muscle;fallback_muscle;fallback_muscle | 4 | False | True | 37 |  |
| H S16 EC | HC | EC | ok |  | 0 | 7 | ICLabel:muscle artifact:0.901 | 1 | False | False | 36 |  |
| H S17 EC | HC | EC | ok |  | 0 | 7,10,11,12 | fallback_muscle;fallback_muscle;fallback_muscle;fallback_muscle | 4 | False | True | 38 |  |
| H S19 EC | HC | EC | ok | Fp1 | 1 | 3,5 | ICLabel:eye blink:0.975;ICLabel:eye blink:0.985 | 2 | False | False | 37 |  |
| H S2 EC | HC | EC | ok |  | 0 |  |  | 0 | False | False | 37 |  |
| H S20 EC | HC | EC | ok |  | 0 | 9 | ICLabel:eye blink:0.997 | 1 | False | False | 38 |  |
| H S21 EC | HC | EC | ok |  | 0 | 14 | fallback_muscle | 1 | False | True | 37 |  |
| H S22 EC | HC | EC | ok |  | 0 | 6 | fallback_muscle | 1 | False | True | 37 |  |
| H S23 EC | HC | EC | ok |  | 0 |  |  | 0 | False | False | 37 |  |
| H S24 EC | HC | EC | ok |  | 0 | 1,4 | fallback_EOG;fallback_EOG | 2 | True | False | 37 |  |
| H S25 EC | HC | EC | ok |  | 0 |  |  | 0 | False | False | 37 |  |
| H S26 EC | HC | EC | ok |  | 0 | 11 | fallback_muscle | 1 | False | True | 37 |  |
| H S27 EC | HC | EC | ok |  | 0 |  |  | 0 | False | False | 37 |  |
| H S28 EC | HC | EC | ok |  | 0 | 3,9 | ICLabel:muscle artifact:0.970;ICLabel:muscle artifact:0.911 | 2 | False | False | 37 |  |
| H S29 EC | HC | EC | ok |  | 0 | 3,7 | ICLabel:eye blink:0.916;ICLabel:eye blink:0.983 | 2 | False | False | 37 |  |
| H S3 EC | HC | EC | ok |  | 0 | 9 | ICLabel:eye blink:0.957 | 1 | False | False | 37 |  |
| H S30 EC | HC | EC | ok |  | 0 |  |  | 0 | False | False | 37 |  |
| H S4 EC | HC | EC | ok |  | 0 | 10 | fallback_muscle | 1 | False | True | 37 |  |
| H S5 EC | HC | EC | ok |  | 0 | 9 | ICLabel:eye blink:0.973 | 1 | False | False | 37 |  |
| H S6 EC | HC | EC | ok |  | 0 | 4 | fallback_muscle | 1 | False | True | 37 |  |
| H S7 EC | HC | EC | ok |  | 0 | 0,3 | ICLabel:eye blink:0.966;ICLabel:eye blink:0.988 | 2 | False | False | 44 |  |
| H S8 EC | HC | EC | ok |  | 0 | 2 | ICLabel:eye blink:1.000 | 1 | False | False | 37 |  |
| H S9 EC | HC | EC | ok |  | 0 |  |  | 0 | False | False | 37 |  |
| MDD S1 EC | MDD | EC | ok | C4,Cz,F8,O2,P4,P8,Pz,T8 | 8 |  |  | 0 | False | False | 37 |  |
| MDD S10 EC | MDD | EC | ok |  | 0 |  |  | 0 | False | False | 37 |  |
| MDD S11 EC | MDD | EC | ok |  | 0 | 4,5 | ICLabel:eye blink:0.996;ICLabel:muscle artifact:0.991 | 2 | False | False | 37 |  |
| MDD S13 EC | MDD | EC | ok |  | 0 | 1,7 | ICLabel:eye blink:0.936;ICLabel:eye blink:0.946 | 2 | False | False | 37 |  |
| MDD S14 EC | MDD | EC | ok |  | 0 | 6 | ICLabel:eye blink:0.946 | 1 | False | False | 37 |  |
| MDD S15 EC | MDD | EC | ok | Cz,F8,P4,Pz,T8 | 5 |  |  | 0 | False | False | 37 |  |
| MDD S17 EC | MDD | EC | ok |  | 0 | 5 | ICLabel:eye blink:0.919 | 1 | False | False | 37 |  |
| MDD S18 EC | MDD | EC | ok |  | 0 | 9 | ICLabel:eye blink:0.986 | 1 | False | False | 37 |  |
| MDD S19 EC | MDD | EC | ok |  | 0 |  |  | 0 | False | False | 37 |  |
| MDD S2 EC | MDD | EC | ok |  | 0 |  |  | 0 | False | False | 37 |  |
| MDD S20 EC | MDD | EC | ok |  | 0 |  |  | 0 | False | False | 37 |  |
| MDD S21 EC | MDD | EC | ok |  | 0 | 3,4 | ICLabel:muscle artifact:0.972;ICLabel:eye blink:0.964 | 2 | False | False | 37 |  |
| MDD S22 EC | MDD | EC | ok |  | 0 | 0 | ICLabel:eye blink:0.984 | 1 | False | False | 37 |  |
| MDD S23 EC | MDD | EC | ok |  | 0 |  |  | 0 | False | False | 37 |  |
| MDD S24 EC | MDD | EC | ok |  | 0 | 8 | ICLabel:eye blink:0.950 | 1 | False | False | 37 |  |
| MDD S25 EC | MDD | EC | ok |  | 0 |  |  | 0 | False | False | 37 |  |
| MDD S26 EC | MDD | EC | ok | Cz,P8,T8 | 3 | 5 | ICLabel:eye blink:0.990 | 1 | False | False | 37 |  |
| MDD S27 EC | MDD | EC | ok |  | 0 | 11 | fallback_muscle | 1 | False | True | 37 |  |
| MDD S28 EC | MDD | EC | ok | Fp2 | 1 |  |  | 0 | False | False | 37 |  |
| MDD S29 EC | MDD | EC | ok |  | 0 |  |  | 0 | False | False | 37 |  |
| MDD S3 EC | MDD | EC | ok |  | 0 | 6 | ICLabel:eye blink:0.976 | 1 | False | False | 22 |  |
| MDD S30 EC | MDD | EC | ok |  | 0 | 11 | ICLabel:muscle artifact:0.948 | 1 | False | False | 30 |  |
| MDD S31 EC | MDD | EC | ok |  | 0 | 7 | ICLabel:eye blink:0.979 | 1 | False | False | 37 |  |
| MDD S32 EC | MDD | EC | ok |  | 0 |  |  | 0 | False | False | 37 |  |
| MDD S33 EC | MDD | EC | ok |  | 0 | 8 | ICLabel:eye blink:0.958 | 1 | False | False | 37 |  |
| MDD S34 EC | MDD | EC | ok |  | 0 | 8 | ICLabel:eye blink:0.958 | 1 | False | False | 37 |  |
| MDD S5 EC | MDD | EC | ok |  | 0 | 6 | ICLabel:eye blink:0.945 | 1 | False | False | 37 |  |
| MDD S6 EC | MDD | EC | ok |  | 0 | 4,10 | ICLabel:eye blink:0.991;ICLabel:eye blink:0.996 | 2 | False | False | 37 |  |
| MDD S7 EC | MDD | EC | ok | Fp1,Fp2 | 2 |  |  | 0 | False | False | 37 |  |
| MDD S9 EC | MDD | EC | ok |  | 0 |  |  | 0 | False | False | 37 |  |

**Supplementary Table S2. Robust edge-wise statistical results underlying Figure 3.**

Only edges surviving both Holm-corrected two-sided Brunner-Munzel testing and Westfall-Young maxT confirmation are listed. Direction and Hedges' g are expressed as MDD minus healthy controls. Edge-level n values reflect finite observations retained after outlier handling; therefore, n can vary slightly by edge.

| **Band** | **Estimator** | **Edge** | **Direction** | **HC n** | **MDD n** | **HC mean** | **MDD mean** | **MDD-HC mean diff.** | **Hedges' g** | **Brunner-Munzel p** | **Holm-adjusted p** | **Westfall-Young maxT p** |
| --- | --- | --- | --- | --- | --- | --- | --- | --- | --- | --- | --- | --- |
| Theta | Coherence | Fp1--O2 | MDD < HC | 28 | 25 | 0.300300 | 0.100430 | -0.199873 | -1.323519 | 0.000000 | 2.100e-05 | 0.000200 |
| Theta | Coherence | T7--O2 | MDD < HC | 27 | 26 | 0.288900 | 0.123394 | -0.165479 | -1.345140 | 1.000e-06 | 9.100e-05 | 0.000200 |
| Theta | Coherence | P8--O2 | MDD < HC | 28 | 26 | 0.381400 | 0.147852 | -0.233561 | -1.312401 | 2.000e-06 | 0.000378 | 0.000200 |
| Theta | Coherence | F8--C3 | MDD < HC | 27 | 25 | 0.225500 | 0.105419 | -0.120051 | -1.287366 | 1.000e-06 | 0.000230 | 0.000400 |
| Theta | Coherence | F3--C4 | MDD < HC | 28 | 29 | 0.293300 | 0.118172 | -0.175086 | -1.278645 | 1.900e-05 | 0.002971 | 0.000400 |
| Theta | Coherence | P4--O2 | MDD < HC | 28 | 30 | 0.452400 | 0.247754 | -0.204635 | -1.158818 | 1.000e-06 | 0.000141 | 0.003199 |
| Theta | Coherence | Fp2--T7 | MDD < HC | 28 | 27 | 0.310000 | 0.152753 | -0.157289 | -1.162744 | 6.000e-05 | 0.009468 | 0.003199 |
| Theta | Coherence | F7--Cz | MDD < HC | 28 | 29 | 0.437100 | 0.247444 | -0.189632 | -1.169548 | 6.700e-05 | 0.010480 | 0.003199 |
| Theta | Coherence | F7--P8 | MDD < HC | 28 | 25 | 0.274100 | 0.103335 | -0.170730 | -1.107425 | 0.000171 | 0.025604 | 0.004799 |
| Theta | Coherence | Fp1--P8 | MDD < HC | 28 | 30 | 0.335600 | 0.166122 | -0.169464 | -1.134927 | 0.000177 | 0.026368 | 0.005399 |
| Theta | Coherence | P4--T7 | MDD < HC | 28 | 23 | 0.250700 | 0.091871 | -0.158862 | -1.077181 | 7.000e-06 | 0.001106 | 0.006399 |
| Theta | Coherence | F4--C3 | MDD < HC | 27 | 28 | 0.247300 | 0.142106 | -0.105145 | -1.135977 | 4.000e-06 | 0.000685 | 0.006599 |
| Theta | Coherence | C3--O1 | MDD < HC | 27 | 27 | 0.240800 | 0.125388 | -0.115413 | -1.133804 | 3.000e-06 | 0.000567 | 0.006999 |
| Theta | Coherence | F7--C4 | MDD < HC | 28 | 27 | 0.247800 | 0.100270 | -0.147482 | -1.105032 | 4.000e-06 | 0.000727 | 0.006999 |
| Theta | Coherence | Pz--O2 | MDD < HC | 28 | 29 | 0.327800 | 0.174327 | -0.153440 | -1.109157 | 9.000e-06 | 0.001391 | 0.006999 |
| Theta | Coherence | C4--O2 | MDD < HC | 28 | 26 | 0.323800 | 0.136905 | -0.186853 | -1.094807 | 5.500e-05 | 0.008723 | 0.006999 |
| Theta | Coherence | F4--P8 | MDD < HC | 28 | 28 | 0.292700 | 0.154283 | -0.138389 | -1.063707 | 2.500e-05 | 0.004006 | 0.011398 |
| Theta | Coherence | T8--O1 | MDD < HC | 28 | 27 | 0.283300 | 0.135592 | -0.147715 | -1.043987 | 0.000132 | 0.019996 | 0.012797 |
| Theta | Coherence | F4--O2 | MDD < HC | 28 | 25 | 0.262600 | 0.108975 | -0.153614 | -1.027198 | 0.000226 | 0.033397 | 0.012797 |
| Theta | Coherence | Fp2--Pz | MDD < HC | 27 | 25 | 0.244400 | 0.110690 | -0.133679 | -1.041169 | 1.800e-05 | 0.002935 | 0.016397 |
| Theta | Coherence | P3--O1 | MDD < HC | 28 | 29 | 0.476000 | 0.316642 | -0.159352 | -1.005934 | 0.000118 | 0.017990 | 0.020196 |
| Theta | Coherence | T7--P7 | MDD < HC | 28 | 29 | 0.417700 | 0.228815 | -0.188930 | -1.009184 | 0.000313 | 0.046015 | 0.020596 |
| Theta | Coherence | C3--O2 | MDD < HC | 28 | 29 | 0.261300 | 0.125898 | -0.135428 | -0.996471 | 0.000119 | 0.018045 | 0.023795 |
| Theta | Coherence | Fz--P8 | MDD < HC | 28 | 30 | 0.302900 | 0.160632 | -0.142255 | -0.979873 | 0.000335 | 0.048631 | 0.027195 |
| Theta | Coherence | F3--C3 | MDD < HC | 28 | 29 | 0.340100 | 0.217553 | -0.122543 | -0.940098 | 0.000106 | 0.016501 | 0.039592 |
| Theta | Coherence | C3--P8 | MDD < HC | 28 | 27 | 0.234400 | 0.115417 | -0.118957 | -0.909269 | 0.000114 | 0.017576 | 0.048390 |
| Theta | Coherence | Fz--P7 | MDD < HC | 28 | 29 | 0.294200 | 0.156800 | -0.137359 | -0.911340 | 0.000320 | 0.046655 | 0.048990 |
| Theta | PLV | C3--O1 | MDD < HC | 28 | 28 | 0.419100 | 0.234745 | -0.184339 | -1.445823 | 0.000000 | 0.000000 | 0.000200 |
| Theta | PLV | P4--O2 | MDD < HC | 28 | 30 | 0.699400 | 0.456413 | -0.243003 | -1.469558 | 0.000000 | 0.000000 | 0.000200 |
| Theta | PLV | P3--O1 | MDD < HC | 28 | 30 | 0.773900 | 0.558718 | -0.215172 | -1.377102 | 0.000000 | 4.000e-06 | 0.000400 |
| Theta | PLV | F7--Cz | MDD < HC | 28 | 30 | 0.657600 | 0.423949 | -0.233688 | -1.369575 | 1.000e-06 | 0.000103 | 0.000400 |
| Theta | PLV | Fp2--P3 | MDD < HC | 28 | 27 | 0.358000 | 0.184038 | -0.173998 | -1.288274 | 3.400e-05 | 0.005217 | 0.001000 |
| Theta | PLV | Fp1--O2 | MDD < HC | 28 | 26 | 0.439900 | 0.230016 | -0.209875 | -1.286641 | 3.500e-05 | 0.005451 | 0.001000 |
| Theta | PLV | P4--T7 | MDD < HC | 28 | 26 | 0.348900 | 0.191582 | -0.157309 | -1.257513 | 7.000e-06 | 0.001093 | 0.001400 |
| Theta | PLV | F3--C4 | MDD < HC | 28 | 29 | 0.451200 | 0.251409 | -0.199749 | -1.270740 | 1.600e-05 | 0.002464 | 0.001800 |
| Theta | PLV | F4--C3 | MDD < HC | 28 | 30 | 0.418300 | 0.273725 | -0.144538 | -1.210823 | 0.000000 | 8.100e-05 | 0.002599 |
| Theta | PLV | Fp2--T7 | MDD < HC | 28 | 30 | 0.486400 | 0.313453 | -0.172978 | -1.212232 | 2.000e-06 | 0.000303 | 0.002799 |
| Theta | PLV | Fp2--F3 | MDD < HC | 28 | 30 | 0.583400 | 0.378636 | -0.204793 | -1.186415 | 9.000e-06 | 0.001499 | 0.002999 |
| Theta | PLV | F8--C3 | MDD < HC | 28 | 30 | 0.366300 | 0.234736 | -0.131535 | -1.188033 | 1.000e-05 | 0.001556 | 0.003799 |
| Theta | PLV | F8--Pz | MDD < HC | 28 | 29 | 0.418700 | 0.229990 | -0.188694 | -1.161672 | 6.000e-05 | 0.009010 | 0.006599 |
| Theta | PLV | F7--C4 | MDD < HC | 28 | 28 | 0.370400 | 0.219697 | -0.150706 | -1.147062 | 1.400e-05 | 0.002270 | 0.007199 |
| Theta | PLV | Fp2--Pz | MDD < HC | 28 | 30 | 0.386900 | 0.223736 | -0.163115 | -1.153710 | 1.000e-05 | 0.001650 | 0.007598 |
| Theta | PLV | Fp1--Cz | MDD < HC | 28 | 30 | 0.520900 | 0.340497 | -0.180363 | -1.141338 | 1.400e-05 | 0.002270 | 0.007798 |
| Theta | PLV | Fp2--F4 | MDD < HC | 27 | 30 | 0.831400 | 0.676037 | -0.155319 | -1.087296 | 8.000e-06 | 0.001391 | 0.008798 |
| Theta | PLV | Fp1--O1 | MDD < HC | 28 | 28 | 0.422200 | 0.236440 | -0.185781 | -1.122965 | 0.000113 | 0.016228 | 0.008798 |
| Theta | PLV | Pz--O2 | MDD < HC | 28 | 30 | 0.496900 | 0.331065 | -0.165805 | -1.103478 | 9.000e-06 | 0.001514 | 0.010198 |
| Theta | PLV | F7--P8 | MDD < HC | 28 | 29 | 0.423200 | 0.229705 | -0.193468 | -1.118836 | 4.000e-05 | 0.006069 | 0.010198 |
| Theta | PLV | Fp1--P8 | MDD < HC | 28 | 30 | 0.492700 | 0.297600 | -0.195083 | -1.099802 | 0.000157 | 0.022156 | 0.012198 |
| Theta | PLV | F3--F7 | MDD > HC | 28 | 30 | 0.674500 | 0.821440 | 0.146941 | 1.089524 | 0.000213 | 0.029347 | 0.014197 |
| Theta | PLV | F4--P8 | MDD < HC | 28 | 29 | 0.435100 | 0.284053 | -0.151060 | -1.080281 | 1.800e-05 | 0.002734 | 0.015397 |
| Theta | PLV | C4--P8 | MDD < HC | 28 | 30 | 0.458700 | 0.317944 | -0.140796 | -1.068348 | 4.800e-05 | 0.007237 | 0.016397 |
| Theta | PLV | Fz--P7 | MDD < HC | 28 | 28 | 0.456100 | 0.273127 | -0.182971 | -1.069547 | 7.600e-05 | 0.011032 | 0.017197 |
| Theta | PLV | C3--O2 | MDD < HC | 28 | 30 | 0.365600 | 0.234633 | -0.130939 | -1.064243 | 6.500e-05 | 0.009685 | 0.018396 |
| Theta | PLV | Cz--Pz | MDD < HC | 28 | 30 | 0.692600 | 0.540472 | -0.152162 | -1.042154 | 0.000232 | 0.031569 | 0.019396 |
| Theta | PLV | Fp1--C3 | MDD < HC | 28 | 25 | 0.396200 | 0.257684 | -0.138522 | -1.049291 | 4.800e-05 | 0.007178 | 0.019996 |
| Theta | PLV | F7--Fz | MDD < HC | 28 | 29 | 0.451200 | 0.280113 | -0.171076 | -1.053662 | 7.100e-05 | 0.010417 | 0.019996 |
| Theta | PLV | C4--T7 | MDD < HC | 28 | 30 | 0.390700 | 0.258592 | -0.132075 | -1.035912 | 0.000123 | 0.017491 | 0.022396 |
| Theta | PLV | T7--O2 | MDD < HC | 28 | 30 | 0.436500 | 0.281881 | -0.154603 | -1.027590 | 4.700e-05 | 0.007178 | 0.023795 |
| Theta | PLV | T8--O1 | MDD < HC | 28 | 30 | 0.443800 | 0.268999 | -0.174849 | -1.032193 | 0.000233 | 0.031569 | 0.024595 |
| Theta | PLV | T7--P7 | MDD < HC | 28 | 30 | 0.610400 | 0.407256 | -0.203147 | -1.007761 | 0.000115 | 0.016481 | 0.028594 |
| Theta | PLV | F4--T7 | MDD < HC | 28 | 30 | 0.380600 | 0.228175 | -0.152410 | -1.021116 | 0.000167 | 0.023175 | 0.028994 |
| Theta | PLV | Fz--C3 | MDD < HC | 28 | 29 | 0.361800 | 0.264773 | -0.097059 | -0.987252 | 7.100e-05 | 0.010417 | 0.034993 |
| Theta | PLV | P8--O1 | MDD < HC | 28 | 30 | 0.491300 | 0.330767 | -0.160560 | -0.966133 | 0.000161 | 0.022557 | 0.039392 |
| Theta | PLV | F3--T8 | MDD < HC | 28 | 29 | 0.415400 | 0.317837 | -0.097575 | -0.949307 | 0.000219 | 0.030040 | 0.048790 |
| Theta | PLI | T8--P8 | MDD < HC | 28 | 27 | 0.256000 | 0.139619 | -0.116391 | -1.759781 | 0.000000 | 0.000000 | 0.000200 |
| Theta | PLI | P8--O1 | MDD < HC | 28 | 27 | 0.271300 | 0.154897 | -0.116452 | -1.503071 | 0.000000 | 0.000000 | 0.000200 |
| Theta | PLI | F7--F8 | MDD < HC | 28 | 27 | 0.264400 | 0.139397 | -0.125043 | -1.422935 | 0.000000 | 1.000e-06 | 0.000200 |
| Theta | PLI | F3--F8 | MDD < HC | 28 | 29 | 0.277400 | 0.162545 | -0.114857 | -1.330232 | 0.000000 | 3.800e-05 | 0.000200 |
| Theta | PLI | F4--Cz | MDD < HC | 28 | 28 | 0.299000 | 0.180151 | -0.118802 | -1.334376 | 1.000e-06 | 0.000115 | 0.000200 |
| Theta | PLI | P8--O2 | MDD < HC | 28 | 27 | 0.295700 | 0.167820 | -0.127861 | -1.423675 | 3.000e-06 | 0.000473 | 0.000200 |
| Theta | PLI | Fp2--P8 | MDD < HC | 28 | 27 | 0.274400 | 0.157002 | -0.117371 | -1.376469 | 4.000e-06 | 0.000600 | 0.000200 |
| Theta | PLI | F8--P4 | MDD < HC | 28 | 22 | 0.243200 | 0.132148 | -0.111036 | -1.270658 | 6.000e-06 | 0.000967 | 0.000200 |
| Theta | PLI | Fp2--T8 | MDD < HC | 28 | 29 | 0.279000 | 0.160664 | -0.118317 | -1.438299 | 1.100e-05 | 0.001626 | 0.000200 |
| Theta | PLI | Fp2--C3 | MDD < HC | 28 | 28 | 0.271300 | 0.150367 | -0.120889 | -1.429182 | 1.600e-05 | 0.002436 | 0.000200 |
| Theta | PLI | C4--O2 | MDD < HC | 28 | 25 | 0.269700 | 0.145733 | -0.124001 | -1.298721 | 3.500e-05 | 0.005062 | 0.000200 |
| Theta | PLI | C3--T8 | MDD < HC | 28 | 26 | 0.272900 | 0.152417 | -0.120482 | -1.330399 | 3.800e-05 | 0.005364 | 0.000200 |
| Theta | PLI | Fp1--F7 | MDD < HC | 28 | 27 | 0.287000 | 0.164570 | -0.122385 | -1.334520 | 0.000158 | 0.020992 | 0.000200 |
| Theta | PLI | C3--O2 | MDD < HC | 28 | 26 | 0.257300 | 0.130291 | -0.126968 | -1.306354 | 0.000000 | 6.000e-06 | 0.000400 |
| Theta | PLI | P4--T8 | MDD < HC | 28 | 27 | 0.260200 | 0.148464 | -0.111694 | -1.314000 | 2.000e-06 | 0.000333 | 0.000400 |
| Theta | PLI | P4--Pz | MDD < HC | 28 | 26 | 0.259900 | 0.152749 | -0.107142 | -1.276484 | 0.000000 | 3.800e-05 | 0.000600 |
| Theta | PLI | Fz--P3 | MDD < HC | 28 | 25 | 0.254700 | 0.147071 | -0.107670 | -1.258224 | 1.000e-06 | 0.000134 | 0.000600 |
| Theta | PLI | F3--T8 | MDD < HC | 28 | 29 | 0.261100 | 0.149345 | -0.111716 | -1.285554 | 1.000e-06 | 0.000144 | 0.000600 |
| Theta | PLI | C3--C4 | MDD < HC | 28 | 26 | 0.244100 | 0.133398 | -0.110684 | -1.285235 | 3.200e-05 | 0.004741 | 0.000600 |
| Theta | PLI | F8--T7 | MDD < HC | 28 | 28 | 0.256200 | 0.155521 | -0.100712 | -1.263703 | 0.000000 | 7.200e-05 | 0.000800 |
| Theta | PLI | T8--O2 | MDD < HC | 28 | 26 | 0.260400 | 0.153114 | -0.107271 | -1.251035 | 3.000e-06 | 0.000491 | 0.000800 |
| Theta | PLI | F8--P8 | MDD < HC | 28 | 27 | 0.275600 | 0.156433 | -0.119132 | -1.238708 | 3.500e-05 | 0.005053 | 0.000800 |
| Theta | PLI | Fp1--P3 | MDD < HC | 28 | 25 | 0.264900 | 0.137278 | -0.127644 | -1.214733 | 3.500e-05 | 0.005062 | 0.000800 |
| Theta | PLI | P4--O2 | MDD < HC | 28 | 27 | 0.285000 | 0.169138 | -0.115844 | -1.237972 | 6.500e-05 | 0.009262 | 0.000800 |
| Theta | PLI | F4--O2 | MDD < HC | 28 | 29 | 0.280900 | 0.159434 | -0.121455 | -1.245662 | 2.700e-05 | 0.004018 | 0.001000 |
| Theta | PLI | Fp1--Fp2 | MDD < HC | 28 | 29 | 0.286100 | 0.166867 | -0.119184 | -1.220756 | 0.000155 | 0.020873 | 0.001600 |
| Theta | PLI | T7--P8 | MDD < HC | 28 | 27 | 0.259900 | 0.143850 | -0.116055 | -1.202685 | 9.000e-06 | 0.001311 | 0.001800 |
| Theta | PLI | F7--P8 | MDD < HC | 28 | 28 | 0.244300 | 0.146147 | -0.098198 | -1.214366 | 6.600e-05 | 0.009310 | 0.001800 |
| Theta | PLI | F8--Cz | MDD < HC | 28 | 29 | 0.267800 | 0.162155 | -0.105621 | -1.212633 | 2.000e-06 | 0.000347 | 0.002200 |
| Theta | PLI | Fp2--P3 | MDD < HC | 28 | 28 | 0.276400 | 0.147802 | -0.128557 | -1.205920 | 1.100e-05 | 0.001626 | 0.002200 |
| Theta | PLI | Fp2--Pz | MDD < HC | 28 | 29 | 0.273300 | 0.150161 | -0.123116 | -1.212263 | 0.000345 | 0.043432 | 0.002200 |
| Theta | PLI | F8--Fz | MDD < HC | 28 | 28 | 0.263500 | 0.169550 | -0.093999 | -1.197350 | 6.600e-05 | 0.009310 | 0.002400 |
| Theta | PLI | F4--Pz | MDD < HC | 28 | 28 | 0.286500 | 0.167771 | -0.118733 | -1.195990 | 9.800e-05 | 0.013519 | 0.002400 |
| Theta | PLI | P4--P8 | MDD < HC | 28 | 28 | 0.287300 | 0.183241 | -0.104075 | -1.182891 | 1.500e-05 | 0.002297 | 0.002599 |
| Theta | PLI | P4--O1 | MDD < HC | 28 | 28 | 0.248800 | 0.152443 | -0.096348 | -1.180216 | 7.700e-05 | 0.010713 | 0.002599 |
| Theta | PLI | F8--O1 | MDD < HC | 28 | 27 | 0.244100 | 0.152292 | -0.091816 | -1.144620 | 0.000251 | 0.032846 | 0.003199 |
| Theta | PLI | C3--Pz | MDD < HC | 28 | 25 | 0.249800 | 0.138099 | -0.111663 | -1.110173 | 0.000332 | 0.042475 | 0.003599 |
| Theta | PLI | F8--P7 | MDD < HC | 28 | 29 | 0.241700 | 0.148286 | -0.093444 | -1.128560 | 0.000155 | 0.020873 | 0.004599 |
| Theta | PLI | C4--O1 | MDD < HC | 28 | 27 | 0.250700 | 0.143098 | -0.107590 | -1.114247 | 9.000e-06 | 0.001338 | 0.005199 |
| Theta | PLI | F3--Cz | MDD < HC | 28 | 27 | 0.277600 | 0.176595 | -0.101048 | -1.113828 | 0.000119 | 0.016357 | 0.005199 |
| Theta | PLI | Fp1--C3 | MDD < HC | 28 | 29 | 0.273500 | 0.184395 | -0.089113 | -1.099974 | 3.000e-06 | 0.000410 | 0.005799 |
| Theta | PLI | P7--O1 | MDD < HC | 28 | 27 | 0.266000 | 0.170762 | -0.095244 | -1.099383 | 0.000307 | 0.039856 | 0.005799 |
| Theta | PLI | Cz--Pz | MDD < HC | 28 | 30 | 0.274000 | 0.172118 | -0.101841 | -1.077455 | 0.000135 | 0.018318 | 0.008598 |
| Theta | PLI | P3--O1 | MDD < HC | 28 | 29 | 0.253600 | 0.158432 | -0.095178 | -1.029780 | 0.000313 | 0.040354 | 0.014597 |
| Theta | PLI | P7--P8 | MDD < HC | 28 | 28 | 0.275500 | 0.175914 | -0.099622 | -1.018326 | 0.000362 | 0.045189 | 0.016197 |
| Theta | PLI | Fp2--F7 | MDD < HC | 28 | 29 | 0.289300 | 0.173067 | -0.116192 | -1.020821 | 0.000400 | 0.049632 | 0.016197 |
| Theta | PLI | F8--Pz | MDD < HC | 28 | 27 | 0.232900 | 0.154065 | -0.078873 | -1.011910 | 0.000192 | 0.025349 | 0.016797 |
| Theta | PLI | F7--Cz | MDD < HC | 28 | 28 | 0.251800 | 0.162297 | -0.089495 | -0.968770 | 0.000332 | 0.042475 | 0.025395 |
| Theta | PLI | Pz--O2 | MDD < HC | 28 | 30 | 0.296600 | 0.190488 | -0.106115 | -0.965182 | 0.000404 | 0.049749 | 0.026195 |
| Theta | MI | Fp2--F8 | MDD > HC | 25 | 29 | 0.017200 | 0.032986 | 0.015774 | 1.237097 | 4.000e-06 | 0.000621 | 0.001400 |
| Theta | MI | F3--T7 | MDD > HC | 27 | 29 | 0.014300 | 0.024448 | 0.010167 | 1.125507 | 5.000e-06 | 0.000913 | 0.005799 |
| Theta | MI | F7--T7 | MDD > HC | 25 | 28 | 0.019800 | 0.033393 | 0.013563 | 0.997430 | 5.500e-05 | 0.009218 | 0.035793 |
| Alpha | Coherence | C4--O2 | MDD < HC | 27 | 28 | 0.332500 | 0.196110 | -0.136408 | -1.473076 | 0.000000 | 7.000e-06 | 0.000200 |
| Alpha | Coherence | F8--Cz | MDD < HC | 28 | 28 | 0.368600 | 0.175228 | -0.193382 | -1.267832 | 4.000e-06 | 0.000605 | 0.001800 |
| Alpha | Coherence | Fp1--Cz | MDD < HC | 26 | 26 | 0.279000 | 0.152578 | -0.126419 | -1.196545 | 0.000000 | 3.500e-05 | 0.005599 |
| Alpha | Coherence | Fp2--C4 | MDD < HC | 27 | 28 | 0.282500 | 0.167420 | -0.115065 | -1.162690 | 1.000e-06 | 0.000233 | 0.005999 |
| Alpha | Coherence | F7--Fz | MDD < HC | 27 | 28 | 0.253300 | 0.167552 | -0.085759 | -1.146668 | 2.800e-05 | 0.004573 | 0.006999 |
| Alpha | Coherence | F7--Cz | MDD < HC | 27 | 29 | 0.372800 | 0.197499 | -0.175306 | -1.130435 | 2.100e-05 | 0.003426 | 0.008798 |
| Alpha | Coherence | Cz--O2 | MDD < HC | 27 | 29 | 0.375600 | 0.258343 | -0.117264 | -1.108410 | 5.000e-05 | 0.008257 | 0.009398 |
| Alpha | Coherence | Fp2--F3 | MDD < HC | 28 | 29 | 0.310000 | 0.187617 | -0.122364 | -1.028997 | 0.000285 | 0.046222 | 0.025395 |
| Alpha | Coherence | Cz--O1 | MDD < HC | 28 | 28 | 0.382300 | 0.256981 | -0.125308 | -1.026111 | 0.000158 | 0.025747 | 0.025595 |
| Alpha | PLV | Fp2--C4 | MDD < HC | 28 | 29 | 0.504800 | 0.301649 | -0.203139 | -1.522180 | 0.000000 | 0.000000 | 0.000200 |
| Alpha | PLV | Fp1--Cz | MDD < HC | 28 | 30 | 0.515300 | 0.306614 | -0.208687 | -1.397374 | 0.000000 | 3.000e-06 | 0.000400 |
| Alpha | PLV | F8--Fz | MDD < HC | 28 | 29 | 0.468500 | 0.285594 | -0.182897 | -1.411064 | 0.000000 | 4.000e-06 | 0.000400 |
| Alpha | PLV | F7--Cz | MDD < HC | 28 | 30 | 0.594500 | 0.329744 | -0.264788 | -1.407060 | 0.000000 | 1.800e-05 | 0.000400 |
| Alpha | PLV | F8--Cz | MDD < HC | 28 | 29 | 0.565800 | 0.317870 | -0.247925 | -1.406462 | 1.000e-06 | 0.000246 | 0.000400 |
| Alpha | PLV | Fz--P3 | MDD < HC | 28 | 24 | 0.473100 | 0.308976 | -0.164123 | -1.313195 | 1.100e-05 | 0.001788 | 0.000800 |
| Alpha | PLV | F4--P4 | MDD < HC | 28 | 30 | 0.480200 | 0.307105 | -0.173107 | -1.299525 | 4.000e-06 | 0.000610 | 0.001600 |
| Alpha | PLV | Cz--O2 | MDD < HC | 28 | 30 | 0.605800 | 0.429128 | -0.176712 | -1.272530 | 1.000e-06 | 0.000187 | 0.001800 |
| Alpha | PLV | C4--O2 | MDD < HC | 28 | 30 | 0.552900 | 0.382737 | -0.170211 | -1.277974 | 2.000e-06 | 0.000259 | 0.001800 |
| Alpha | PLV | F7--Fz | MDD < HC | 28 | 30 | 0.444100 | 0.286880 | -0.157218 | -1.219690 | 4.000e-06 | 0.000703 | 0.002799 |
| Alpha | PLV | C3--P8 | MDD < HC | 28 | 28 | 0.374200 | 0.263576 | -0.110582 | -1.172876 | 1.000e-05 | 0.001663 | 0.004799 |
| Alpha | PLV | Fp2--F3 | MDD < HC | 28 | 30 | 0.513000 | 0.335738 | -0.177271 | -1.162624 | 1.000e-05 | 0.001594 | 0.005199 |
| Alpha | PLV | F3--F4 | MDD < HC | 28 | 29 | 0.439400 | 0.277564 | -0.161801 | -1.162633 | 2.900e-05 | 0.004601 | 0.005799 |
| Alpha | PLV | Fp1--P3 | MDD < HC | 28 | 29 | 0.415400 | 0.270002 | -0.145428 | -1.109381 | 8.000e-05 | 0.012253 | 0.011998 |
| Alpha | PLV | Fz--P8 | MDD < HC | 28 | 30 | 0.502300 | 0.352589 | -0.149757 | -1.087542 | 7.100e-05 | 0.011095 | 0.014397 |
| Alpha | PLV | C3--O2 | MDD < HC | 28 | 28 | 0.422600 | 0.248243 | -0.174344 | -1.092263 | 7.500e-05 | 0.011699 | 0.014997 |
| Alpha | PLV | Fp1--O2 | MDD < HC | 28 | 28 | 0.403200 | 0.250711 | -0.152534 | -1.064974 | 0.000124 | 0.018791 | 0.018396 |
| Alpha | PLV | F4--F7 | MDD < HC | 28 | 28 | 0.394200 | 0.269678 | -0.124493 | -1.035942 | 4.000e-05 | 0.006270 | 0.028594 |
| Alpha | PLV | Cz--P8 | MDD < HC | 28 | 30 | 0.567300 | 0.435745 | -0.131505 | -1.027554 | 0.000109 | 0.016527 | 0.029594 |
| Alpha | PLV | Fz--T8 | MDD < HC | 28 | 30 | 0.495200 | 0.337637 | -0.157539 | -1.025919 | 0.000197 | 0.029279 | 0.033793 |
| Alpha | PLV | Fp2--Cz | MDD < HC | 27 | 30 | 0.443300 | 0.309109 | -0.134211 | -1.033172 | 7.400e-05 | 0.011515 | 0.033993 |
| Alpha | PLV | F4--T8 | MDD < HC | 28 | 30 | 0.406200 | 0.293790 | -0.112390 | -1.005383 | 0.000322 | 0.047399 | 0.038192 |
| Alpha | PLV | Fp1--C4 | MDD < HC | 28 | 30 | 0.409000 | 0.272651 | -0.136334 | -0.992140 | 8.000e-05 | 0.012253 | 0.043991 |
| Alpha | PLV | P3--O2 | MDD < HC | 28 | 30 | 0.414800 | 0.291418 | -0.123365 | -0.988474 | 0.000154 | 0.023090 | 0.044591 |
| Alpha | PLI | F7--F8 | MDD < HC | 28 | 24 | 0.241200 | 0.150049 | -0.091124 | -1.134303 | 8.800e-05 | 0.014996 | 0.005799 |
| Alpha | PLI | F3--F8 | MDD < HC | 28 | 29 | 0.232500 | 0.166430 | -0.066079 | -1.092301 | 0.000147 | 0.024966 | 0.016197 |
| Alpha | MI | F7--T7 | MDD > HC | 25 | 30 | 0.016600 | 0.033761 | 0.017155 | 1.161225 | 0.000000 | 1.800e-05 | 0.002999 |
| Alpha | MI | Fp1--Fz | MDD > HC | 23 | 29 | 0.017700 | 0.032005 | 0.014260 | 1.147738 | 3.000e-06 | 0.000577 | 0.005399 |
| Alpha | MI | F3--T7 | MDD > HC | 26 | 29 | 0.012300 | 0.024051 | 0.011768 | 1.146293 | 2.000e-05 | 0.003399 | 0.005399 |
| Alpha | MI | F8--T8 | MDD > HC | 24 | 29 | 0.016700 | 0.036965 | 0.020307 | 1.064163 | 3.100e-05 | 0.005158 | 0.010598 |
| Alpha | MI | Fp1--T7 | MDD > HC | 27 | 30 | 0.013200 | 0.024853 | 0.011664 | 1.062756 | 5.600e-05 | 0.009314 | 0.011598 |
| Alpha | MI | Cz--O1 | MDD < HC | 27 | 28 | 0.039300 | 0.022012 | -0.017329 | -1.006228 | 0.000239 | 0.039510 | 0.046591 |
| Beta | Coherence | O1--O2 | MDD < HC | 27 | 25 | 0.301000 | 0.139310 | -0.161738 | -1.301294 | 0.000000 | 8.000e-05 | 0.001000 |
| Beta | PLV | O1--O2 | MDD < HC | 28 | 30 | 0.552600 | 0.277654 | -0.274943 | -1.512053 | 1.000e-06 | 0.000140 | 0.000400 |
| Beta | PLV | T8--P7 | MDD < HC | 28 | 29 | 0.490500 | 0.274287 | -0.216214 | -1.229678 | 1.100e-05 | 0.001821 | 0.002200 |
| Beta | PLV | F3--C4 | MDD < HC | 28 | 28 | 0.415900 | 0.213457 | -0.202435 | -1.153911 | 0.000189 | 0.031295 | 0.005199 |
| Beta | PLV | F7--C4 | MDD < HC | 27 | 26 | 0.290700 | 0.192814 | -0.097921 | -1.104082 | 3.900e-05 | 0.006551 | 0.017197 |
| Beta | PLV | F7--O1 | MDD < HC | 28 | 26 | 0.440300 | 0.246086 | -0.194179 | -1.076456 | 7.900e-05 | 0.013163 | 0.017596 |
| Beta | PLV | Fp2--O1 | MDD < HC | 28 | 28 | 0.269300 | 0.197206 | -0.072092 | -1.028259 | 3.100e-05 | 0.005162 | 0.035593 |
| Beta | PLI | Fp2--C3 | MDD < HC | 27 | 27 | 0.173800 | 0.138242 | -0.035567 | -1.078931 | 2.100e-05 | 0.003507 | 0.021996 |
| Beta | MI | F3--T7 | MDD > HC | 23 | 30 | 0.005100 | 0.023581 | 0.018459 | 1.561658 | 0.000000 | 0.000000 | 0.000200 |
| Beta | MI | F3--P7 | MDD > HC | 23 | 29 | 0.005700 | 0.020533 | 0.014803 | 1.532901 | 0.000000 | 0.000000 | 0.000200 |
| Beta | MI | C3--P7 | MDD > HC | 23 | 30 | 0.006000 | 0.019894 | 0.013909 | 1.563764 | 0.000000 | 0.000000 | 0.000200 |
| Beta | MI | Fp1--P7 | MDD > HC | 19 | 29 | 0.005100 | 0.019947 | 0.014853 | 1.425298 | 0.000000 | 0.000000 | 0.000200 |
| Beta | MI | Fz--T7 | MDD > HC | 22 | 30 | 0.005100 | 0.018902 | 0.013827 | 1.447730 | 0.000000 | 0.000000 | 0.000200 |
| Beta | MI | F7--T7 | MDD > HC | 22 | 30 | 0.010800 | 0.032991 | 0.022185 | 1.332505 | 0.000000 | 0.000000 | 0.000200 |
| Beta | MI | F3--O1 | MDD > HC | 23 | 29 | 0.003600 | 0.010193 | 0.006633 | 1.393930 | 0.000000 | 0.000000 | 0.000200 |
| Beta | MI | F7--P7 | MDD > HC | 23 | 30 | 0.007500 | 0.025023 | 0.017558 | 1.362317 | 0.000000 | 0.000000 | 0.000200 |
| Beta | MI | F4--P7 | MDD > HC | 22 | 29 | 0.004300 | 0.012369 | 0.008087 | 1.411078 | 0.000000 | 5.300e-05 | 0.000200 |
| Beta | MI | Fp1--T7 | MDD > HC | 24 | 29 | 0.006800 | 0.022946 | 0.016115 | 1.232788 | 0.000000 | 1.000e-06 | 0.000400 |
| Beta | MI | F3--P3 | MDD > HC | 23 | 30 | 0.006500 | 0.016109 | 0.009577 | 1.246769 | 0.000000 | 1.000e-06 | 0.000400 |
| Beta | MI | F7--O1 | MDD > HC | 23 | 30 | 0.005100 | 0.014118 | 0.009023 | 1.296667 | 0.000000 | 9.000e-06 | 0.000400 |
| Beta | MI | F3--C3 | MDD > HC | 24 | 30 | 0.007600 | 0.022665 | 0.015099 | 1.231219 | 0.000000 | 1.400e-05 | 0.000400 |
| Beta | MI | C3--O1 | MDD > HC | 23 | 29 | 0.004600 | 0.010115 | 0.005545 | 1.288000 | 0.000000 | 2.300e-05 | 0.000400 |
| Beta | MI | Fz--C4 | MDD > HC | 23 | 30 | 0.007800 | 0.019959 | 0.012191 | 1.210143 | 0.000000 | 3.800e-05 | 0.000400 |
| Beta | MI | Pz--O1 | MDD > HC | 23 | 28 | 0.006200 | 0.012179 | 0.005998 | 1.243962 | 0.000000 | 4.400e-05 | 0.000400 |
| Beta | MI | C4--P7 | MDD > HC | 24 | 30 | 0.005300 | 0.013574 | 0.008259 | 1.274186 | 3.000e-06 | 0.000413 | 0.000400 |
| Beta | MI | Fz--O1 | MDD > HC | 23 | 29 | 0.004400 | 0.010189 | 0.005820 | 1.240884 | 2.100e-05 | 0.002897 | 0.000400 |
| Beta | MI | Fz--P7 | MDD > HC | 26 | 30 | 0.007600 | 0.017878 | 0.010248 | 1.264981 | 2.500e-05 | 0.003425 | 0.000400 |
| Beta | MI | C3--Pz | MDD > HC | 23 | 30 | 0.005600 | 0.015288 | 0.009694 | 1.229647 | 2.900e-05 | 0.003918 | 0.000400 |
| Beta | MI | Fp1--C3 | MDD > HC | 21 | 30 | 0.005000 | 0.017605 | 0.012593 | 1.141570 | 0.000000 | 2.000e-06 | 0.000600 |
| Beta | MI | F4--T7 | MDD > HC | 23 | 30 | 0.005000 | 0.013835 | 0.008844 | 1.165001 | 0.000000 | 3.900e-05 | 0.000800 |
| Beta | MI | P3--O2 | MDD > HC | 22 | 29 | 0.006200 | 0.011756 | 0.005527 | 1.194058 | 0.000000 | 5.100e-05 | 0.000800 |
| Beta | MI | Fp1--C4 | MDD > HC | 23 | 30 | 0.005700 | 0.015597 | 0.009894 | 1.166692 | 1.000e-06 | 0.000121 | 0.000800 |
| Beta | MI | Fp1--Fz | MDD > HC | 24 | 28 | 0.009100 | 0.021235 | 0.012130 | 1.229461 | 3.000e-06 | 0.000418 | 0.000800 |
| Beta | MI | Pz--P7 | MDD > HC | 26 | 30 | 0.007000 | 0.015903 | 0.008931 | 1.170215 | 2.300e-05 | 0.003121 | 0.001000 |
| Beta | MI | Fp1--F4 | MDD > HC | 25 | 30 | 0.008100 | 0.021287 | 0.013166 | 1.127847 | 0.000000 | 5.000e-05 | 0.001200 |
| Beta | MI | F7--C3 | MDD > HC | 25 | 30 | 0.006800 | 0.019675 | 0.012852 | 1.159377 | 1.000e-06 | 0.000187 | 0.001200 |
| Beta | MI | Fp2--P7 | MDD > HC | 21 | 30 | 0.005800 | 0.014810 | 0.008982 | 1.108143 | 0.000176 | 0.021068 | 0.001200 |
| Beta | MI | F8--O1 | MDD > HC | 23 | 30 | 0.004600 | 0.010625 | 0.006061 | 1.116513 | 0.000195 | 0.022843 | 0.001200 |
| Beta | MI | F7--P3 | MDD > HC | 23 | 29 | 0.005400 | 0.011628 | 0.006237 | 1.152821 | 5.000e-06 | 0.000721 | 0.001600 |
| Beta | MI | C4--O1 | MDD > HC | 23 | 30 | 0.005400 | 0.010619 | 0.005211 | 1.127292 | 4.500e-05 | 0.005896 | 0.001600 |
| Beta | MI | Fz--O2 | MDD > HC | 23 | 30 | 0.004400 | 0.010129 | 0.005762 | 1.091714 | 0.000414 | 0.045937 | 0.001600 |
| Beta | MI | F4--F8 | MDD > HC | 25 | 29 | 0.014500 | 0.034799 | 0.020322 | 1.145702 | 5.000e-06 | 0.000710 | 0.001800 |
| Beta | MI | Fp2--F8 | MDD > HC | 23 | 30 | 0.008800 | 0.027285 | 0.018529 | 1.091999 | 1.900e-05 | 0.002693 | 0.001800 |
| Beta | MI | F4--F7 | MDD > HC | 26 | 29 | 0.007100 | 0.016915 | 0.009830 | 1.137628 | 2.000e-06 | 0.000326 | 0.002999 |
| Beta | MI | C3--O2 | MDD > HC | 23 | 30 | 0.003800 | 0.008951 | 0.005130 | 1.070150 | 1.300e-05 | 0.001891 | 0.002999 |
| Beta | MI | C4--T7 | MDD > HC | 23 | 28 | 0.005000 | 0.012004 | 0.007043 | 1.128762 | 3.000e-06 | 0.000463 | 0.003199 |
| Beta | MI | P3--P8 | MDD > HC | 23 | 29 | 0.005500 | 0.010407 | 0.004891 | 1.122501 | 0.000337 | 0.038046 | 0.003199 |
| Beta | MI | Fp2--F7 | MDD > HC | 23 | 30 | 0.010600 | 0.029082 | 0.018473 | 1.064373 | 0.000190 | 0.022623 | 0.003999 |
| Beta | MI | Cz--P7 | MDD > HC | 22 | 30 | 0.007900 | 0.018444 | 0.010496 | 1.071345 | 1.400e-05 | 0.001994 | 0.004599 |
| Beta | MI | Cz--P3 | MDD > HC | 24 | 29 | 0.007200 | 0.013703 | 0.006525 | 1.109117 | 0.000170 | 0.020721 | 0.004599 |
| Beta | MI | C4--Pz | MDD > HC | 25 | 30 | 0.006700 | 0.015120 | 0.008402 | 1.073324 | 6.600e-05 | 0.008346 | 0.004799 |
| Beta | MI | Fz--Pz | MDD > HC | 25 | 30 | 0.005500 | 0.013117 | 0.007599 | 1.062437 | 0.000297 | 0.034112 | 0.004799 |
| Beta | MI | Fp1--P3 | MDD > HC | 22 | 29 | 0.004500 | 0.009596 | 0.005058 | 1.074150 | 3.000e-05 | 0.003955 | 0.005199 |
| Beta | MI | F8--P7 | MDD > HC | 23 | 29 | 0.005900 | 0.013989 | 0.008103 | 1.060377 | 3.600e-05 | 0.004755 | 0.005799 |
| Beta | MI | P7--O2 | MDD > HC | 23 | 30 | 0.007400 | 0.013850 | 0.006462 | 1.045838 | 0.000192 | 0.022686 | 0.005999 |
| Beta | MI | F7--Pz | MDD > HC | 25 | 30 | 0.004900 | 0.011306 | 0.006377 | 1.023355 | 0.000446 | 0.048783 | 0.007798 |
| Beta | MI | F4--O1 | MDD > HC | 24 | 30 | 0.004200 | 0.009102 | 0.004889 | 1.006770 | 0.000305 | 0.034735 | 0.007998 |
| Beta | MI | Fp1--F8 | MDD > HC | 25 | 29 | 0.010700 | 0.024243 | 0.013552 | 1.030394 | 3.600e-05 | 0.004765 | 0.010598 |
| Beta | MI | C3--T7 | MDD > HC | 27 | 30 | 0.013300 | 0.029511 | 0.016257 | 1.019354 | 5.900e-05 | 0.007568 | 0.011998 |
| Beta | MI | F3--F7 | MDD > HC | 28 | 29 | 0.025500 | 0.050141 | 0.024605 | 1.038526 | 5.100e-05 | 0.006571 | 0.012398 |
| Beta | MI | F3--Cz | MDD > HC | 25 | 30 | 0.006500 | 0.017017 | 0.010549 | 0.971682 | 0.000168 | 0.020635 | 0.014597 |
| Beta | MI | F8--T8 | MDD > HC | 22 | 27 | 0.008600 | 0.026211 | 0.017562 | 1.013446 | 7.000e-06 | 0.000931 | 0.016597 |
| Beta | MI | P4--P7 | MDD > HC | 24 | 29 | 0.005600 | 0.010779 | 0.005176 | 0.984759 | 0.000197 | 0.022843 | 0.017996 |
| Beta | MI | Fp2--Fz | MDD > HC | 24 | 30 | 0.011300 | 0.023031 | 0.011725 | 0.955902 | 0.000443 | 0.048783 | 0.021596 |
| Beta | MI | Pz--O2 | MDD > HC | 24 | 28 | 0.006200 | 0.011208 | 0.004983 | 0.988311 | 0.000112 | 0.013991 | 0.022196 |
| Beta | MI | F3--T8 | MDD > HC | 25 | 27 | 0.004900 | 0.011754 | 0.006851 | 1.001815 | 3.900e-05 | 0.005154 | 0.023995 |
| Beta | MI | F8--Fz | MDD > HC | 26 | 29 | 0.010100 | 0.021134 | 0.011024 | 0.966294 | 8.500e-05 | 0.010680 | 0.024195 |
| Beta | MI | Fp1--F7 | MDD > HC | 25 | 28 | 0.012000 | 0.028131 | 0.016139 | 0.980929 | 2.800e-05 | 0.003754 | 0.025595 |
| Beta | MI | P7--O1 | MDD > HC | 25 | 28 | 0.009700 | 0.016673 | 0.006998 | 0.997392 | 0.000153 | 0.018940 | 0.027195 |
| Beta | MI | P3--T7 | MDD > HC | 26 | 29 | 0.008600 | 0.014795 | 0.006170 | 0.954684 | 0.000171 | 0.020721 | 0.037992 |

**Supplementary Table S3. Graph-theoretical statistical results underlying Figure 4.**

Weighted undirected sensor-level graphs were constructed from subject-level 19 x 19 connectivity matrices after retaining the strongest 20% of edges. Graph metrics were compared between MDD and healthy-control groups using two-sided Brunner-Munzel tests. Holm correction was applied across graph measures within each band x estimator family. Hedges' g is expressed as MDD minus healthy controls.

| **Band** | **Estimator** | **Graph metric** | **HC n** | **MDD n** | **HC mean** | **MDD mean** | **MDD-HC mean diff.** | **Hedges' g** | **Brunner-Munzel statistic** | **Brunner-Munzel p** | **Holm-adjusted p** | **Holm significant** |
| --- | --- | --- | --- | --- | --- | --- | --- | --- | --- | --- | --- | --- |
| Theta | Coherence | Average clustering | 28 | 30 | 0.198409 | 0.173249 | -0.025160 | -0.266391 | 1.772561 | 0.082020 | 0.164040 | No |
| Theta | Coherence | Mean weighted strength | 28 | 30 | 1.806612 | 1.476336 | -0.330277 | -0.633067 | 2.743544 | 0.008199 | 0.032795 | Yes |
| Theta | Coherence | Betweenness centrality | 28 | 30 | 0.070900 | 0.074005 | 0.003105 | 0.155091 | -0.452964 | 0.652331 | 0.652331 | No |
| Theta | Coherence | Global efficiency | 28 | 30 | 0.231192 | 0.189123 | -0.042069 | -0.825973 | 2.965216 | 0.004622 | 0.023109 | Yes |
| Theta | Coherence | Local efficiency | 28 | 30 | 0.180743 | 0.151359 | -0.029384 | -0.281631 | 2.029170 | 0.047452 | 0.142356 | No |
| Theta | PLV | Average clustering | 28 | 30 | 0.216860 | 0.172628 | -0.044232 | -0.635737 | 2.183620 | 0.033852 | 0.069760 | No |
| Theta | PLV | Mean weighted strength | 28 | 30 | 2.698640 | 2.337661 | -0.360979 | -1.194447 | 4.130927 | 0.000140 | 0.000594 | Yes |
| Theta | PLV | Betweenness centrality | 28 | 30 | 0.077092 | 0.078913 | 0.001821 | 0.132528 | -0.176690 | 0.860404 | 0.860404 | No |
| Theta | PLV | Global efficiency | 28 | 30 | 0.368830 | 0.322799 | -0.046032 | -1.199034 | 4.181590 | 0.000119 | 0.000594 | Yes |
| Theta | PLV | Local efficiency | 28 | 30 | 0.249396 | 0.194992 | -0.054404 | -0.648238 | 2.341992 | 0.023253 | 0.069760 | No |
| Theta | PLI | Average clustering | 28 | 30 | 0.186030 | 0.146176 | -0.039854 | -0.697319 | 2.715354 | 0.008790 | 0.017580 | Yes |
| Theta | PLI | Mean weighted strength | 28 | 30 | 1.261465 | 0.947356 | -0.314109 | -0.988862 | 4.949433 | 8.347e-06 | 4.174e-05 | Yes |
| Theta | PLI | Betweenness centrality | 28 | 30 | 0.057669 | 0.062573 | 0.004904 | 0.382433 | -1.546059 | 0.127737 | 0.127737 | No |
| Theta | PLI | Global efficiency | 28 | 30 | 0.167294 | 0.130149 | -0.037146 | -0.805518 | 3.653319 | 0.000573 | 0.001720 | Yes |
| Theta | PLI | Local efficiency | 28 | 30 | 0.113187 | 0.065777 | -0.047410 | -1.170302 | 4.961287 | 8.644e-06 | 4.174e-05 | Yes |
| Theta | MI | Average clustering | 28 | 30 | 0.140598 | 0.162089 | 0.021492 | 0.290871 | -1.525031 | 0.132889 | 0.664446 | No |
| Theta | MI | Mean weighted strength | 28 | 30 | 0.214170 | 0.167246 | -0.046924 | -0.440464 | 1.304996 | 0.197777 | 0.677470 | No |
| Theta | MI | Betweenness centrality | 28 | 30 | 0.075950 | 0.068593 | -0.007357 | -0.205270 | 0.908958 | 0.367283 | 0.677470 | No |
| Theta | MI | Global efficiency | 28 | 30 | 0.025106 | 0.018615 | -0.006491 | -0.535082 | 1.373891 | 0.176444 | 0.677470 | No |
| Theta | MI | Local efficiency | 28 | 30 | 0.027886 | 0.021656 | -0.006230 | -0.447234 | 1.393842 | 0.169367 | 0.677470 | No |
| Alpha | Coherence | Average clustering | 28 | 30 | 0.210092 | 0.199311 | -0.010781 | -0.127168 | 0.557938 | 0.579260 | 1.000000 | No |
| Alpha | Coherence | Mean weighted strength | 28 | 30 | 1.737931 | 1.519304 | -0.218627 | -0.511418 | 3.025843 | 0.003787 | 0.015148 | Yes |
| Alpha | Coherence | Betweenness centrality | 28 | 30 | 0.076662 | 0.078523 | 0.001861 | 0.085166 | -0.174968 | 0.861818 | 1.000000 | No |
| Alpha | Coherence | Global efficiency | 28 | 30 | 0.233426 | 0.195672 | -0.037754 | -1.086983 | 4.588849 | 3.051e-05 | 0.000153 | Yes |
| Alpha | Coherence | Local efficiency | 28 | 30 | 0.178152 | 0.167634 | -0.010518 | -0.114509 | 0.761195 | 0.450000 | 1.000000 | No |
| Alpha | PLV | Average clustering | 28 | 30 | 0.233897 | 0.199295 | -0.034601 | -0.479906 | 1.866542 | 0.067373 | 0.135124 | No |
| Alpha | PLV | Mean weighted strength | 28 | 30 | 2.646360 | 2.356758 | -0.289602 | -1.132282 | 4.437233 | 5.118e-05 | 0.000205 | Yes |
| Alpha | PLV | Betweenness centrality | 28 | 30 | 0.078554 | 0.081550 | 0.002996 | 0.224963 | -0.555520 | 0.580809 | 0.580809 | No |
| Alpha | PLV | Global efficiency | 28 | 30 | 0.368199 | 0.327640 | -0.040560 | -1.202639 | 5.141493 | 4.919e-06 | 2.460e-05 | Yes |
| Alpha | PLV | Local efficiency | 28 | 30 | 0.256613 | 0.216346 | -0.040268 | -0.495262 | 2.055978 | 0.045041 | 0.135124 | No |
| Alpha | PLI | Average clustering | 28 | 30 | 0.201298 | 0.188263 | -0.013035 | -0.220535 | 1.481224 | 0.144152 | 0.720760 | No |
| Alpha | PLI | Mean weighted strength | 28 | 30 | 1.376011 | 1.285817 | -0.090194 | -0.276728 | 1.215115 | 0.229615 | 0.918458 | No |
| Alpha | PLI | Betweenness centrality | 28 | 30 | 0.049572 | 0.053228 | 0.003655 | 0.213026 | -1.177125 | 0.244124 | 0.918458 | No |
| Alpha | PLI | Global efficiency | 28 | 30 | 0.174917 | 0.163103 | -0.011814 | -0.281702 | 1.159750 | 0.251300 | 0.918458 | No |
| Alpha | PLI | Local efficiency | 28 | 30 | 0.134490 | 0.127016 | -0.007475 | -0.151533 | 0.891139 | 0.376681 | 0.918458 | No |
| Alpha | MI | Average clustering | 28 | 30 | 0.156968 | 0.168360 | 0.011391 | 0.152421 | -0.564727 | 0.574633 | 1.000000 | No |
| Alpha | MI | Mean weighted strength | 28 | 30 | 0.220582 | 0.191699 | -0.028883 | -0.387007 | 1.480454 | 0.145457 | 0.581829 | No |
| Alpha | MI | Betweenness centrality | 28 | 30 | 0.066957 | 0.066919 | -0.000038 | -0.001579 | 0.053575 | 0.957464 | 1.000000 | No |
| Alpha | MI | Global efficiency | 28 | 30 | 0.026308 | 0.021206 | -0.005102 | -0.598736 | 2.536332 | 0.014905 | 0.074524 | No |
| Alpha | MI | Local efficiency | 28 | 30 | 0.029117 | 0.026008 | -0.003110 | -0.221817 | 0.569082 | 0.572185 | 1.000000 | No |
| Beta | Coherence | Average clustering | 28 | 30 | 0.197083 | 0.164655 | -0.032428 | -0.332803 | 1.594739 | 0.116677 | 0.583385 | No |
| Beta | Coherence | Mean weighted strength | 28 | 30 | 1.494154 | 1.386007 | -0.108147 | -0.204957 | 0.000000 | 1.000000 | 1.000000 | No |
| Beta | Coherence | Betweenness centrality | 28 | 30 | 0.068947 | 0.066873 | -0.002074 | -0.093036 | 0.269339 | 0.788660 | 1.000000 | No |
| Beta | Coherence | Global efficiency | 28 | 30 | 0.188323 | 0.175569 | -0.012754 | -0.300464 | 0.255562 | 0.799684 | 1.000000 | No |
| Beta | Coherence | Local efficiency | 28 | 30 | 0.159548 | 0.139638 | -0.019910 | -0.189873 | 1.109857 | 0.271876 | 1.000000 | No |
| Beta | PLV | Average clustering | 28 | 30 | 0.212320 | 0.154005 | -0.058315 | -0.734868 | 2.607035 | 0.012279 | 0.059276 | No |
| Beta | PLV | Mean weighted strength | 28 | 30 | 2.406459 | 2.150920 | -0.255538 | -0.649939 | 1.545549 | 0.130640 | 0.391920 | No |
| Beta | PLV | Betweenness centrality | 28 | 30 | 0.071797 | 0.070829 | -0.000968 | -0.074043 | 1.105090 | 0.275148 | 0.391920 | No |
| Beta | PLV | Global efficiency | 28 | 30 | 0.328353 | 0.300551 | -0.027802 | -0.589239 | 1.389579 | 0.173135 | 0.391920 | No |
| Beta | PLV | Local efficiency | 28 | 30 | 0.229732 | 0.162851 | -0.066881 | -0.710116 | 2.619654 | 0.011855 | 0.059276 | No |
| Beta | PLI | Average clustering | 28 | 30 | 0.192378 | 0.198224 | 0.005847 | 0.104131 | -0.473235 | 0.638042 | 1.000000 | No |
| Beta | PLI | Mean weighted strength | 28 | 30 | 0.768032 | 0.813376 | 0.045344 | 0.369794 | -1.334913 | 0.188167 | 0.752668 | No |
| Beta | PLI | Betweenness centrality | 28 | 30 | 0.053492 | 0.053343 | -0.000149 | -0.009271 | 0.145830 | 0.884592 | 1.000000 | No |
| Beta | PLI | Global efficiency | 28 | 30 | 0.100908 | 0.103549 | 0.002641 | 0.178477 | -0.415592 | 0.679308 | 1.000000 | No |
| Beta | PLI | Local efficiency | 28 | 30 | 0.070302 | 0.084469 | 0.014167 | 0.481324 | -1.725918 | 0.090379 | 0.451897 | No |
| Beta | MI | Average clustering | 28 | 30 | 0.149708 | 0.185698 | 0.035990 | 0.308761 | -2.274353 | 0.028314 | 0.113255 | No |
| Beta | MI | Mean weighted strength | 28 | 30 | 0.111225 | 0.128694 | 0.017468 | 0.238974 | -1.675469 | 0.099445 | 0.298336 | No |
| Beta | MI | Betweenness centrality | 28 | 30 | 0.070458 | 0.061232 | -0.009226 | -0.270748 | 1.238477 | 0.220783 | 0.441565 | No |
| Beta | MI | Global efficiency | 28 | 30 | 0.012715 | 0.013222 | 0.000507 | 0.069457 | -0.651020 | 0.517698 | 0.517698 | No |
| Beta | MI | Local efficiency | 28 | 30 | 0.014957 | 0.018788 | 0.003831 | 0.315418 | -2.875587 | 0.005945 | 0.029725 | Yes |

**Supplementary Table S4. Comparison of graph-theoretical effect sizes across network-construction approaches**

Values are Hedges’ g (MDD − HC). Negative values indicate lower graph measures in MDD, whereas positive values indicate higher graph measures in MDD.

| **Band–estimator** | **Network construction** | **Mean strength** | **Weighted clustering** | **Global efficiency** | **Local efficiency** | **Betweenness** |
| --- | --- | --- | --- | --- | --- | --- |
| Theta–coherence | 20% proportional | -0.61 | -0.62 | -0.82 | -0.53 | 0.08 |
| Theta–coherence | *Unthresholded weighted* | -0.69 | -0.78 | -0.65 | -0.65 | 0.64 |
| Theta–PLV | 20% proportional | -1.14 | -0.93 | -0.93 | -1.08 | 0.96 |
| Theta–PLV | *Unthresholded weighted* | -1.08 | -0.89 | -1.08 | -1.08 | 0.59 |
| Theta–PLI | 20% proportional | -0.94 | -0.09 | -0.90 | -1.04 | 0.77 |
| Theta–PLI | *Unthresholded weighted* | -0.96 | 0.44 | -0.95 | -0.95 | -0.51 |
| Theta–MI | 20% proportional | -1.08 | -0.80 | -1.11 | -1.06 | 0.14 |
| Theta–MI | *Unthresholded weighted* | -1.02 | 0.42 | -1.04 | -1.04 | -1.09 |
| Alpha–coherence | 20% proportional | -0.48 | -0.38 | -0.78 | -0.34 | 0.34 |
| Alpha–coherence | *Unthresholded weighted* | -0.52 | -0.73 | -0.54 | -0.54 | 0.63 |
| Alpha–PLV | 20% proportional | -1.03 | -0.76 | -0.96 | -1.00 | 0.46 |
| Alpha–PLV | *Unthresholded weighted* | -0.93 | -0.53 | -0.96 | -0.96 | -0.63 |
| Alpha–PLI | 20% proportional | -0.49 | 0.08 | -0.50 | -0.31 | -0.07 |
| Alpha–PLI | *Unthresholded weighted* | -0.52 | 0.08 | -0.52 | -0.52 | -0.43 |
| Alpha–MI | 20% proportional | -0.95 | -0.51 | -0.95 | -0.82 | 0.45 |
| Alpha–MI | *Unthresholded weighted* | -0.85 | 0.30 | -0.92 | -0.92 | -0.96 |
| Beta–coherence | 20% proportional | -0.28 | -0.43 | -0.36 | -0.24 | 0.00 |
| Beta–coherence | *Unthresholded weighted* | -0.32 | -0.44 | -0.33 | -0.33 | 0.25 |
| Beta–PLV | 20% proportional | -0.51 | -1.03 | -0.44 | -0.85 | 0.13 |
| Beta–PLV | *Unthresholded weighted* | -0.61 | -0.62 | -0.58 | -0.59 | 0.88 |
| Beta–PLI | 20% proportional | -0.62 | 0.39 | -0.51 | -0.19 | 0.74 |
| Beta–PLI | *Unthresholded weighted* | -0.62 | 0.10 | -0.63 | -0.63 | -0.68 |
| Beta–MI | 20% proportional | -0.54 | -0.54 | -0.57 | -0.60 | 0.24 |
| Beta–MI | *Unthresholded weighted* | -0.46 | 0.05 | -0.57 | -0.57 | -0.33 |

Abbreviations: PLV, phase-locking value; PLI, phase-lag index; MI, mutual information. The 20% proportional-threshold approach retained the strongest 20% of edges, whereas the unthresholded weighted approach retained the full weighted network.
